# Enantioselective Biomimetic Structures Inspired by Oxi-Dase-Type Metalloenzymes, Utilizing Polynuclear Compounds Containing Copper (II) and Manganese (II) Ions as Building Blocks

**DOI:** 10.3390/biomimetics8050423

**Published:** 2023-09-13

**Authors:** Didier Gómez, Jorge Acosta, Horacio López-Sandoval, Ricardo A. Torres-Palma, Yenny Ávila-Torres

**Affiliations:** 1 Facultad de Tecnologías, Universidad Tecnológica de Pereira, Pereira 660003, Colombia; djgc219@utp.edu.co (D.G.); jlacosta@utp.edu.co (J.A.); 2 Departamento de Química Inorgánica, Facultad de Química, Universidad Nacional Autónoma de México, C.U., Coyoacán, México City 04510, Mexico; balrognazgul@hotmail.com; 3 Grupo de Investigación en Remediación Ambiental y Biocatálisis (GIRAB), Instituto de Química, Facultad de Ciencias Exactas y Naturales, Universidad de Antioquia UdeA, Calle 70 No. 52-21, Medellín 50010, Colombia; ricardo.torres@udea.edu.co

**Keywords:** *ascorbate oxidase*, biomimetics, *catalase*, coordination compounds, electronic effects

## Abstract

This study focuses on developing and evaluating two novel enantioselective biomimetic models for the active centers of oxidases (ascorbate oxidase and catalase). These models aim to serve as alternatives to enzymes, which often have limited action and a delicate nature. For the ascorbate oxidase (AO) model (compound **1**), two enantiomers, S,S(+)cpse and R,R(−)cpse, were combined in a crystalline structure, resulting in a racemic compound. The analysis of their magnetic properties and electrochemical behavior revealed electronic transfer between six metal centers. Compound **1** effectively catalyzed the oxidation of ascorbic to dehydroascorbic acid, showing a 45.5% yield for the racemic form. This was notably higher than the enantiopure compounds synthesized previously and tested in the current report, which exhibited yields of 32% and 28% for the S,S(+)cpse and R,R(-)cpse enantiomers, respectively. This outcome highlights the influence of electronic interactions between metal ions in the racemic compound compared to pure enantiomers. On the other hand, for the catalase model (compound **2**), both the compound and its enantiomer displayed polymeric properties and dimeric behavior in the solid and solution states, respectively. Compound **2** proved to be effective in catalyzing the oxidation of hydrogen peroxide to oxygen with a yield of 64.7%. In contrast, its enantiomer (with R,R(-)cpse) achieved only a 27% yield. This further validates the functional nature of the prepared biomimetic models for oxidases. This research underscores the importance of understanding and designing biomimetic models of metalloenzyme active centers for both biological and industrial applications. These models show promising potential as viable alternatives to natural enzymes in various processes.

## 1. Introduction

Enzymes have garnered significant attention in recent years due to their diverse functionality, serving as biocatalysts. They possess the ability to catalyze various organic reactions, including the creation of C-C bonds. Enzymes’ catalytic properties are influenced by several factors, such as their microenvironment, affinity, reactivity, substrate selectivity, and remarkable recognition capabilities. Despite their valuable characteristics, enzymes face limitations in industrial applications due to their fragility, low thermal stability, and high reactivity with organic solvents and metal ions [1,2,3]. To overcome these challenges while retaining the functional properties of enzymes, biomimetic catalysts have been proposed [4,5]. Polynuclear complexes find utility in numerous applications, and biomimetic oxidases from the oxidoreductive class have the ability to facilitate oxidation–reduction reactions by utilizing dioxygen as an electron acceptor, leading to the production of water or hydrogen peroxide as byproducts [6,7,8]. In contrast, natural enzymes like laccase and ascorbate oxidase (AO) belong to the multicopper oxidase and blue multicopper oxidase categories [9,10]. The enzymes typically possess four copper atoms in their active centers, which actively part water. Furthermore, they have a pivotal function in breaking down detrimental organic contaminants like phenols, chlorophenols, and pharmaceutical compounds. Biocatalytic behavior holds significant value in various biotechnological processes, including the detoxification of effluents, especially in industries like pulp and paper, textiles, and petrochemicals.

**Scheme 1:**
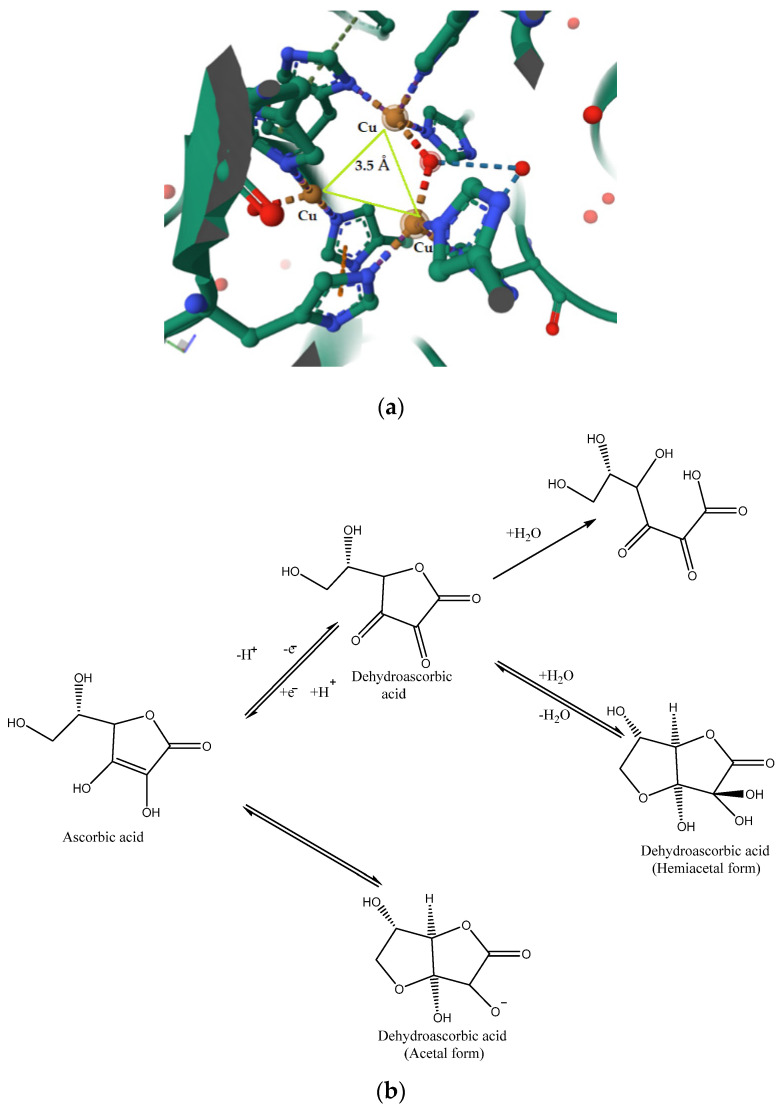
(**a**) Active center for *Ascorbate oxidase* (Messerschmidt et al., 1992), (**b**) the reaction catalyzed by the AO enzyme.

AO, which is being extensively researched, stands as one of the pioneering enzymes to have its structure and active center comprehensively understood [11]. This metalloenzyme operates on L-ascorbic acid (L-AA), which is the most abundant antioxidant present in plant tissues at mmol L^−1^ levels. L-AA can be found in various cellular compartments, including the cytosol, chloroplast, vacuoles, mitochondria, and apoplast. Notably, the amount of L-AA is notably high in the cytosol and chloroplast, ranging between 10 mM and 20 mM, respectively. AO catalyzes the conversion of L-AA and O_2_ into 2-dehydroascorbate (DHA) and water molecules [12]. Subsequently, through a specific carrier transfer process between the oxidized and reduced forms, DHA is transported to the cytosol via the plasma membrane. This ensures that the reduction energy is directed towards the cell wall, as depicted in Scheme 1a. In a similar vein, the active center of ascorbate oxidase, a metalloenzyme, is displayed to enhance understanding of the relationship between its structure and functionality (Scheme 1b). Through spectroscopic and kinetic investigations, it has been discovered that the mononuclear copper and trinuclear copper species originating from the reducing substrate serve as the entry site for electrons and the binding site for dioxygen, respectively. The enzyme operates using a “two-site ping-pong bi-bi” mechanism [13,14].

Manganese-containing enzymes are widely utilized as catalysts in the conversion of compounds, with peroxidases being an example that employs hydrogen peroxide to facilitate reactions [15,16,17] Fungal species have been found to harbor numerous peroxidases. Among the basidiomycetes that colonize wood and cause white rot, manganese peroxidase is the most prevalent peroxidase involved in modifying lignin. This enzyme relies on hydrogen peroxide as a co-substrate to oxidize Mn^2+^ ions present in wood and soil into Mn^3+^ ions (Equation (1)), with stabilization achieved through fungal chelates like oxalic acid [18]. Manganese peroxidase, a plant, and fungal enzymes utilize hydrogen peroxide to oxidize and transform various phenolic substrates, including lignin. Generally, manganese serves as the primary metallic center in the active site of catalases. Its oxidation state can vary depending on the activity, binding of anionic ligands, and charge compensation with other atoms of the same nature (such as iron or manganese). An acidic environment promotes the reduction of Mn^3+^ ions to Mn^2+^ (E° = 1.559 V, Equation (2)), thereby enhancing its oxidative capacity towards substrates like hydrogen peroxide or organic molecules like lignin [19].

Mn_2_^2+^_(ac)_ + H_2_O_2(ac)_ + 2H^+^ → Mn_2_^3+^_(ac)_ + 2H_2_O, (1)

Mn_2_^3+^_(ac)_ + H_2_O_2(ac)_ → Mn_2_^2+^ _(ac)_ + O_2_ + 2H^+^ (2)

This paper describes the design, synthesis, and testing of two new AO and *catalase* biomimetic models as polynuclear enantioselective catalysts. The first compound, [Cu_3_(S,S(+)cpse)_3_(H_2_O)_3_][Cu_3_(R,R(-)cpse)_3_(H_2_O)_3_]·17H_2_O (**1**), has chiral amino alcohols that form a racemic unit in the crystalline cell. This compound also contains a conformed trinuclear arrangement for three cooper atoms similar to the AO active center. This compound was characterized via electronic analyses, magnetism measurements, and electrochemical techniques. The catalytic activities as AO were realized for compound **1** in comparison with the enantiomerically pure compounds synthesized previously [20]. The differences between enantiomers and racemic compounds evidence the enantiomeric effects on the degradation of ascorbic acid, from the viewpoint of the catalyst. In the same manner, compound **2** [Mn_2_(S,S(+)Hcpse)_4_(NaClO_4_)_2_(NaOH)(MeOH)]_n_·[(EtOH)_2_]_n_·[(MeOH)]_n_[H_2_O]_n_ and its enantiomer were synthesized. A crystalline structure was obtained for compound **2** only, and its enantiomeric counterpart was compared using X-ray diffraction patterns in powder, magnetic, electrochemical properties, and circular dichroism in order to recognize its enantiomeric properties. Both were used as a biomimetic model for *oxidase*, observing enantioselective activity in H_2_O_2_ catalysis. This research contributes to the design, evaluation, and comprehension of the enantioselective properties of polynuclear compounds from amino alcohols. This is a significant contribution to understanding activity associated with oxidation processes in these metalloenzymes, which presents two new biomimetic models that can be used in selective industrial processes.

This article presents the development, design, and examination of two novel catalysts that mimic the properties of AO and catalase enzymes. The first compound, denoted as (1), consisted of chiral amino alcohols arranged in a racemic structure within its crystalline unit. It also features a trinuclear configuration of three copper atoms, resembling the active center of AO. Characterization of this compound involved electronic analysis, magnetism measurements, and electrochemical techniques. The catalytic activities of compound **1**, acting as an AO, were compared with previously synthesized [20]. The disparities observed between enantiomers and racemic compounds indicated significant enantiomeric effects on the degradation of ascorbic acid from a catalytic standpoint. Similarly, compound **2**, along with its enantiomer, denoted as (2), was synthesized. Although only the crystalline structure of compound **2** was obtained, a comparison was made between its enantiomeric counterpart using X-ray diffraction patterns in powder form, magnetic and electrochemical properties, as well as circular dichroism. These analyses aimed to identify biomimetic models for oxidase, demonstrating enantioselective activity in catalyzing H_2_O_2_.

This research has made significant contributions to the design, evaluation, and understanding of the enantioselective characteristics of polynuclear compounds derived from amino alcohols. It provides valuable insights into the oxidation processes associated with these metalloenzymes and introduces two biomimetic models that can be effectively employed in selective industrial processes.

## 2. Materials and Methods

### 2.1. Materials

Cu(CH_3_COO)_2_∙H_2_O from Merck and methanol from J.T Baker were utilized without any additional purification. The synthesis of N-[2-hydroxy-1(R)-methyl-2(R)-phenylethyl]-N-methylglycine (R,R(-)H_2_cpse and N-[2-hydroxy-1(S)-methyl-2(S)-phenylethyl]-N-methylglycine (S,S(+)H_2_cpse) ligands followed a procedure described in a prior publication (20).

Mn(CH_3_COO)_2_∙4H_2_O from Merck, NaClO_4_ from J.T. Baker, NaOH from J.T. Baker, and methanol from J.T. Baker were employed without any additional purification. For assessing peroxide and catalase activities, 30% hydrogen peroxide from Aldrich was used.

L-ascorbic acid from Merck, as well as the isomers [Cu_3_(R,R(-)cpse)_3_(H_2_O)_3_]·8.5H_2_O, [Cu_3_(S,S(-)cpse)_3_(H_2_O)_3_]·8.5H_2_O, and precursors [Cu(R,R(-)Hcpse)_2_]∙2H_2_O and [Cu(S,S(+)Hcpse)_2_]∙2H_2_O, were synthesized following a procedure reported in the previous literature [20].

**Scheme 2:**
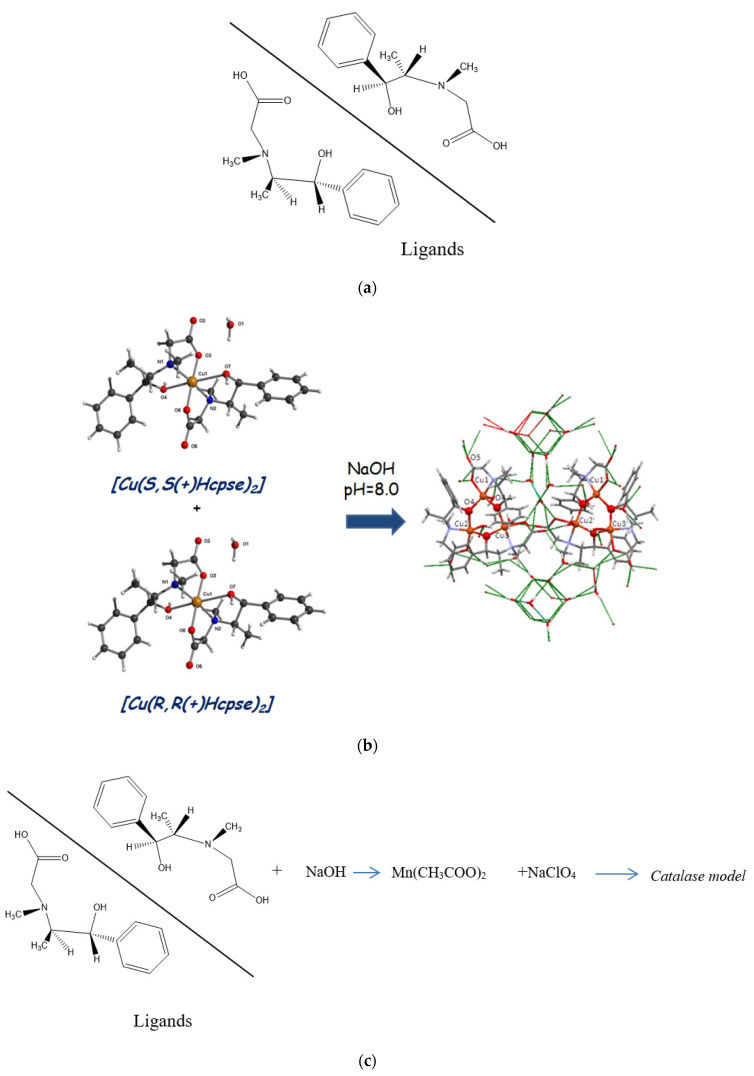
(**a**) Ligands, (**b**) synthesis of *AO biomimetic model,* (**c**) synthesis of *catalase model* [18].

In Scheme 2, the structure of the ligands (Scheme 2a) and their reactions with copper (Scheme 2b) and manganese (Scheme 2c) are shown, respectively.

### 2.2. Synthesis of the Biomimetic Compounds

For the synthesis of compound **1**, mononuclear compounds [Cu(R,R(-)Hcpse)_2_]∙2H_2_O (339 mg, 0.65 mmol) and [Cu(S,S(+)Hcpse)_2_]∙2H_2_O (343 mg, 0.66 mmol) were dissolved in methanol (20 mL) and mixed with 4 equivalents of solid NaOH (104 mg) and Cu(CH_3_COO)_2_∙H_2_O (264 mg, 1.2 mmol). After four weeks, blue crystals suitable for X-ray studies were obtained with a yield of 78.7%. Anal. Calcd. for 2(C_36_H_51_N_3_O_12_Cu_3_)∙17(H_2_O): C% = 40.6; H% = 4.80; N% = 3.96. Found: C% = 39.59; H% = 4.55; N% = 3.85. IR (KBrν/cm^−1^): ν_as_COO^−^ = 1615; ν_s_COO^−^ = 1385 and ∆ν = 230 cm^−1^, Supplementary Table S1 [21].

Compound **2** was synthesized by combining a solution of S,S(+)H_2_cpse (479 mg, 2.14 mmol) in methanol (15 mL) with Mn(CH_3_COO)_2_∙4H_2_O (265 mg, 0.108 mmol). The resulting mixture was stirred for 15 min. Then, NaClO_4_ (13 mg, 0.109 mmol) was added to the mixture, and the pH was adjusted to basic using solid NaOH. The mixture was stirred for an additional 15 min. After a period of two weeks, brown needle-shaped crystals suitable for X-ray diffraction analysis were obtained with a yield of 78%. The compound was hygroscopic. Anal. Calcd for C_54.15_ H_87.30_ Cl_2_Mn_2_N_4_Na_3_O_26_ (2): C%, 43.15, H%, 5.77; N%, 12.84. Found C%, 44.50, %H, 5.98; N% 12.17. IR (KBrν/cm^−1^): 1569(ν_as_ COO^−^) and 1443 (νσ COO^−^) and ∆ν = 126 cm^−1^, SI2. [21]. In order to analyze the stability of the polymer in solution of the polymer, was performedmass spectrometry (MS) was performed and indicated the following peak: m/z [M]^−^, 1322 (dimeric unit (C_48_H_67_Cl_2_Mn_2_N_4_Na_4_O_22_). The m/z peaks were assigned in Supplementary Tables S3 and S4, suggesting a fragmentation on the dimeric unit.

The enantiomeric compound was synthesized in a similar manner to compound **1**, where the R,R(-)H_2_cpse ligand (481 mg, 2.15 mmol) was dissolved in methanol Mn(CH_3_COO)_2_∙4H_2_O (270 mg, 0.11 mmol). The mixture was stirred for 15 min, followed by the addition of NaClO_4_ (15 mg, 01.0 mmol) and adjustment of the pH using solid NaOH. The mixture was stirred for an additional 15 min. After four weeks, brown needle-shaped crystals were observed, but a suitable crystal for single-crystal diffraction was not obtained. In this case, the diffraction patterns of the two isomers were compared to confirm their isostructural relationship [18].

### 2.3. Physical Measurements

In the 4000–400 cm^−1^ range, infrared spectra were captured using a Nicolet Fourier transform infrared (FT-IR) 740 spectrophotometer employing KBr pellets. Elemental analyses were performed using a Fisons EA 1108 elemental analyzer. Electronic spectra (diffuse reflectance) were measured using a Cary 5E UV–visible–near IR (UV–Vis–NIR) spectrophotometer in the range of 250–2500 nm (40000–5000 cm^−1^). Magnetic susceptibility measurements were performed using a pendulum-type magnetometer (MANICS DSM8) equipped with an Oxford CF 1200 s helium continuous-flow cryostat operating at a temperature range of 300 K–4 K in a magnetic field of 3 Oe. Diamagnetic corrections were estimated using Pascal constants. Powder diffraction was recorded on a SIEMENS D500 with graphite-monochromatic Cu-Kα (λ = 1.5406 Å) radiation at 293 K, CT: 0.6 s, SS: 0.020 dg, and WL = 1.5406 Å. Circular dichroism (CD) measurements were conducted using a JASCO J815 spectrophotometer.

### 2.4. X-ray Crystallographic Study

For compound **1**, employing standard protocols, all X-ray diffraction data were gathered using a Nonius Kappa instrument that was equipped with a CCD area detector. The data were obtained using graphite-monochromatic Mo-Kα radiation at a temperature of 293 K. Intensity measurements were taken through j + w scans. The structural analysis followed direct methods using SHELXS-97, and subsequent refinement (utilizing all F2 data) employed full-matrix least-squares techniques within the Crystals 12.84 software. Anisotropic refinement was carried out for all nonhydrogen atoms, whereas hydrogen atom positions were determined geometrically and allowed to be coordinated with their respective atoms. For compound **2**, X-ray diffraction data were acquired using an Agilent Xcalibur Atlas Gemini diffractometer. The data collection utilized graphite-monochromatic Mo-Kα radiation and was performed using ω scans at a temperature of 130 K. A multifaceted crystal-based analytical numeric absorption correction was applied. The structural evaluation for this compound involved direct methods through SHELXS-2012, followed by refinement based on all F^2^ data using SHELXL-2014 and full-matrix least-squares techniques. Certain aspects of the structure exhibited orientation and/or statistical disorder, specifically the perchlorate anions, two phenyl rings (C21 → C26 and C27 → C32), and both coordinated and uncoordinated solvent molecules. Modeling primarily involved two positions to accommodate this disorder. Disordered atoms, with the exception of the solvent methanol molecule (C49-O22), which exhibited non-positive definite displacement parameters and was refined isotropically, were refined with restraints (SIMU and DELU) on anisotropic displacement parameters. The bond geometries of the disordered groups were constrained or restrained to match the AFIX or SADI commands of SHELXL. Additionally, the contribution of smeared uncoordinated solvents was “squeezed” using the PLATON program (as shown in Table 1).

### 2.5. Electrochemical Analyses

Cyclic voltammetry (CV) analyses were performed on copper-based compound **1**, its enantiomeric counterparts, ligands, and precursors. These experiments were carried out in dry, degassed methanol with tetrabutylammonium hexafluorophosphate (TBAPF6) at a concentration of 0.1 M serving as a supporting electrolyte. A CH electrochemical analyzer (CH Instruments, Inc., Bee Cave, TX, USA) was utilized for these measurements. To validate the presence of redox waves, differential pulse voltammetry (DPV) investigations were conducted. The DPV studies utilized specific parameters for the pulse, including an amplitude of 0.020 V, pulse width of 0.050 V, sample width of 0.020 V, and pulse period of 0.100 s. Prior to each experiment, all samples underwent a 5 min degassing process using nitrogen (N2). The concentrations of all compounds were set at 1 mM, and the final volume was 5 mL. For the CV measurements, a scan rate of 100 mV/s was employed, and the samples were cycled between −2.0 V and 2.0 V. The experimental setup included a glassy carbon working electrode, a platinum counter electrode, and an Ag/AgCl reference electrode (with a potential of +0.197 V compared to the normal hydrogen electrode, NHE).

Electrochemical analyses of the manganese-based compound **2** and its enantiomer were performed in a conventional three-electrode cell using a glassy carbon disc (ϕ = 3 mm), platinum mesh, and saturated calomel electrode (SCE) as the working, auxiliary, and reference electrodes, respectively. The supporting electrolyte was n-tetrabutylammonium hexafluorophosphate (n-Bu_4_NPF_6_) (0.1 M). The reference electrode was connected to the working solution through a salt bridge containing the same supporting electrolyte. Distilled acetonitrile was employed as a solvent. All the experiments were performed under an argon atmosphere at room temperature. CV was performed at a scan rate of 0.1 Vs^−1^ unless stated otherwise. All potentials are reported vs. the SCE.

### 2.6. Catalytic Activities of the Biomimetic Models

***AO*** ***biomimetic model:*** The catalytic activity of compound **1** and its enantiomeric compounds were evaluated by reacting 10 ppm of the catalyst with different concentrations of the substrate (L-AA: 300, 600, 900, 1200, and 1500 ppm) in HPLC vials (1.5 mL). The reaction was monitored using an HPLC system (UHPLC-ULTIMATE 3000, Thermo Scientific, Waltham, MA, USA) equipped with a diode array detector (UV–Vis). An analysis was carried out using a Hi-plex-H^®^ column, employing sulfuric acid (0.005 M) as the mobile phase at a flow rate of 0.6 mL/min, and an injection volume of 20 µL. Detection was conducted at varying wavelengths (220, 240, 270, and 300 nm) due to their capability to detect both L-ascorbic acid (L-AA), the substrate, and dehydroascorbic acid (DHA), the end product.

***Catalase biomimetic model:*** The catalytic activity of compound **2** and its enantiomer were evaluated by adding the catalyst (5 ppm) with H_2_O_2_ (50 ppm). The reaction lasted for 60 min, generating oxygen in small bubbles that were deposited in a gas collector. The reaction system consisted of a vessel (cylinder glass, 10 mL capacity) with a cork cap (diameter 17 mm and height 10 mm) fitted to the mouth of the cylinder, an injection system and gas transport system, and a security system to prevent the produced oxygen from diffusing through the cork (a septum stopper with a diameter of 12 mm and a thickness of 3 mm was placed on the outside of the vessel). The determination of hydrogen peroxide degradation was assessed using the iodometry technique. A portion of 600 μL from the reactor was introduced into a quartz cell along with 1350 μL of 0.1 M potassium iodide and 50 μL of 0.01 M ammonium heptamolybdate. At intervals of 5, 10, 15, 20, and 60 min, the absorbance at 350 nm was measured using a Genesys spectrophotometer.

## 3. Results

### 3.1. Crystal Structure of the AO Biomimetic Model

Compound **1** was obtained from a mixture of corresponding enantiomerically pure mononuclear copper(II) compounds, [Cu(R,R(-)Hcpse)_2_]∙2H_2_O and [Cu(S,S(+)Hcpse)_2_]∙2H_2_O. The IR spectra of compound **1** showed that the ν_as_ and ν_s_ stretching modes of the carboxylic group at 1615 cm^−1^ and 1385 cm^−1^ were shifted to relatively low frequencies in its coordination mode, with coordination modes [∆ν(cm^−1^)] at ca. 230 cm^−1^. This shift indicates a monodentate coordination mode. Electronic transitions were not observed in the circular dichroism spectrum of the racemic compound **1**, as expected.

**Figure 1:**
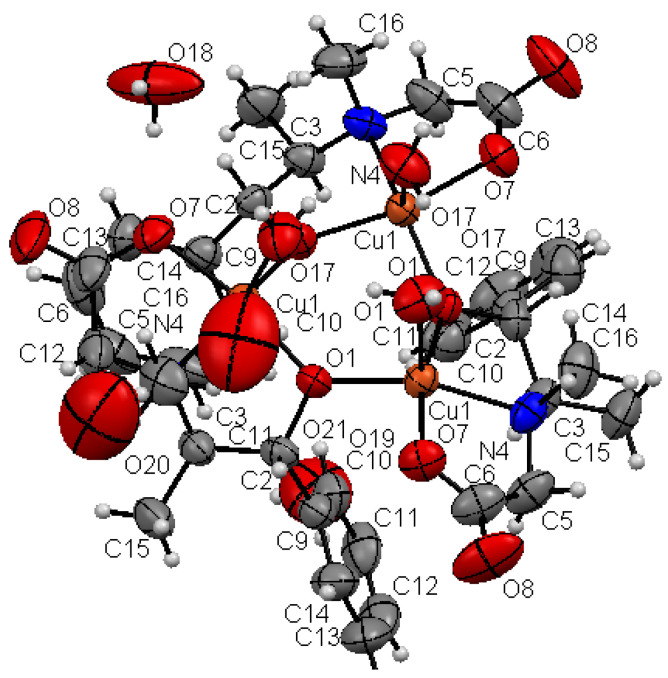
X-ray structure of the copper (II) racemic trinuclear compound (**1**), designation color atom: oxygen atom (red color), carbon atom (grease color), hydrogen atom (white color), copper atom (orange color).

**Figure 2:**
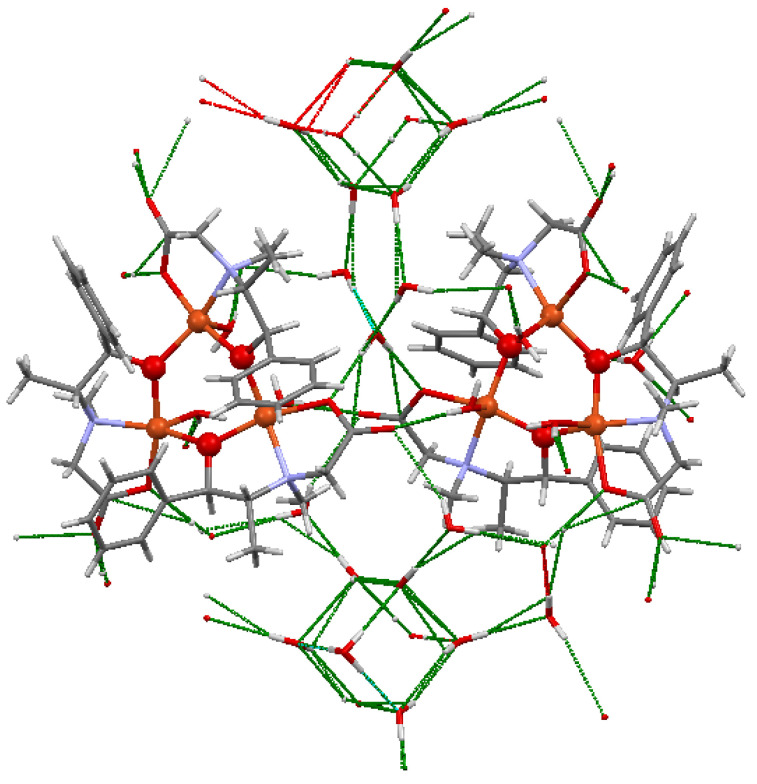
Centered cubic arrangement of the water molecules of compound **1**, intermolecular interactions, designation color atom: oxygen atom (red color), carbon atom (grease color), hydrogen atom (white color), copper atom (orange color).

**Figure 3:**
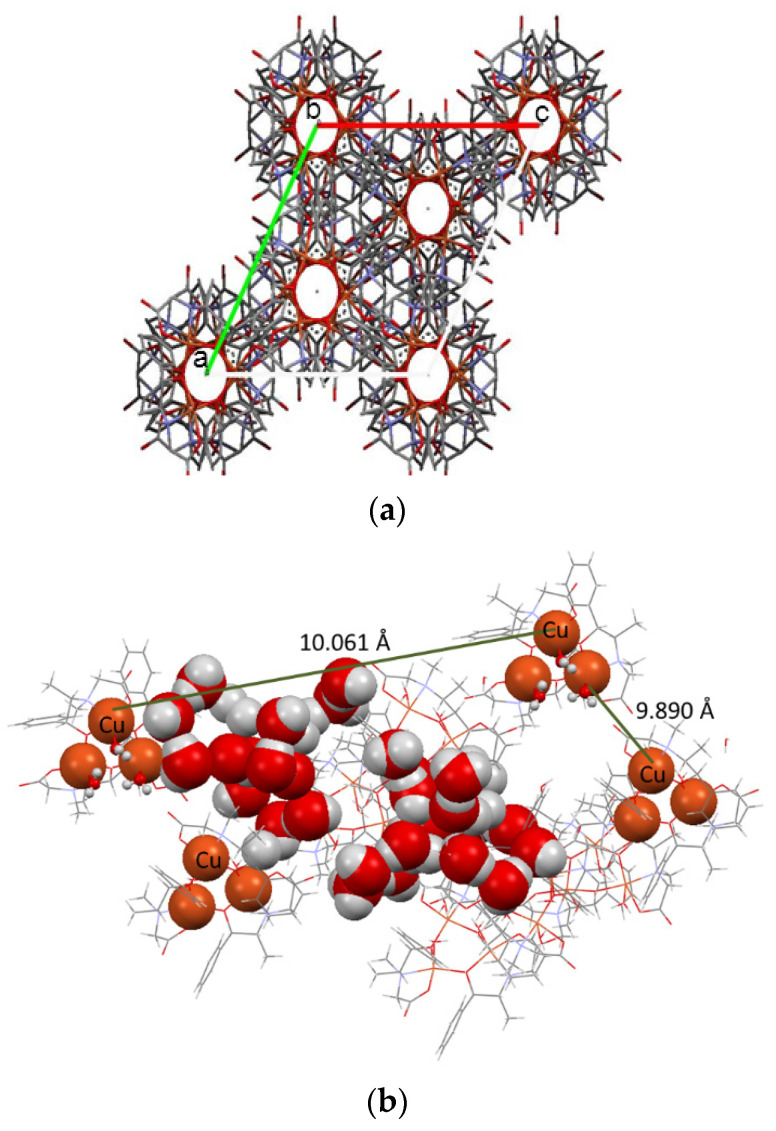
Molecular arrangements showing package in a crystalline cell for compound **1:** (**a**) water molecular arrangement for each enantiomer unit, (**b**) supramolecular arrangements on the c axes; designation color atom: designation atom color: oxygen atom (red color), carbon atom (grease color), hydrogen atom (white color), copper atom (orange color).

The reflectance spectrum of compound **1** displayed an electronic transition centered at 14,636 cm^−1^ (683 nm), indicating a pentacoordinate square-based geometry. The X-ray structure of this compound presents two enantiomer units in a 1:1 relationship with 17 water molecules in the crystalline center-symmetric group (R-3c), forming a racemic compound (Figure 1). The ligands, R,R(-)H_2_cpse and S,S(+)H_2_cpse, are coordinated to the metallic atom and a water molecule in the apical position. Table 2 presents selected angles in the coordination sphere for compound **1**. There are two sets of 17 water molecules between the trinuclear compound units, with a cubic arrangement in the center, where three pentagons are formed on three of its edges, as depicted in (Figure 2). This is an unusual arrangement. Figure 3a shows the corresponding water molecular arrangement for each enantiomer unit. Figure 3b shows the packing in the supramolecular arrangements on the c axes, which exhibits interesting cavities of the water molecules.

**Figure 4:**
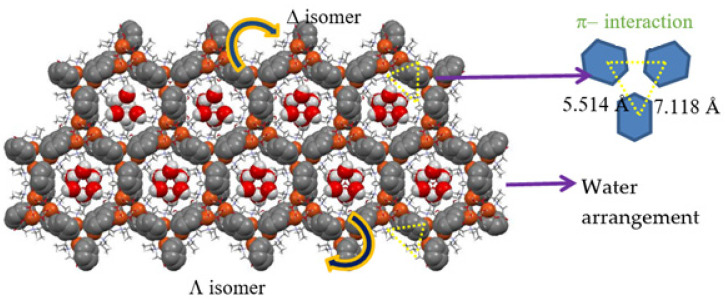
Arrangement of the water molecules of the ∆ and Λ isomers.

The π-stacking interactions for each isomer allow supramolecules with ∆ and Λ isomerism to coexist in the same structure (Figure 4). In this unique arrangement, the water molecules are trapped in the blades, resulting in the stabilization of heptameric polygons. The distances between the centroids corresponding to aromatic rings indicate moderate interactions, which allow this conformation to be achieved. These structures are essential in biological systems where water molecules are outside sphere coordination, as shown in Table 3. Similarly, the binding of water molecules to metals allows labile coordination positions that facilitate biological functions, such as the case of metalloenzymes.

### 3.2. Crystal Structure of the Catalase Biomimetic Model and Spectroscopic Characterization for Its Enaniomeric Corresponding Compound

**Figure 5:**
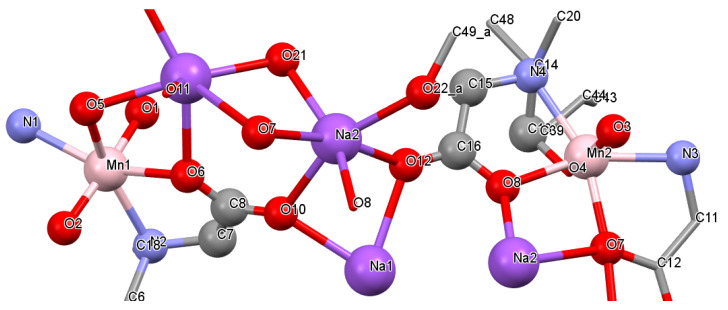
Ligand around metal centers in compound **2**.

The molecular structure of compound **2** features an infinite one-dimensional (1D) coordination polymer constructed from monomeric Mn(S,S(+)Hcpse) units and polymeric {(NaClO_4_)_2_ (NaOH)}*_n_* chains. The geometry around the Mn atoms is a slightly distorted octahedral, and it is formed by two monodeprotonated, bis-chelating, and tri-connected *N*,*O*,*O″*-N-[2-hydroxy-1(*S*)-methyl-2(*S*)-phenylethyl]-N-methylglycinate (S,S(+)H_2_cpse) ligands that adopt a facial arrangement, in which the equatorial plane is formed by the nitrogen atoms, N1(N3) and N2(N4), and the O5(O7) and O6(O8) oxygen atoms form the carboxylic groups, whereas the apical position is occupied by the O1(O3) and O2(O4) hydroxyl groups for the Mn1 and Mn2 atoms, respectively (Figure 5) [22]

**Figure 6:**
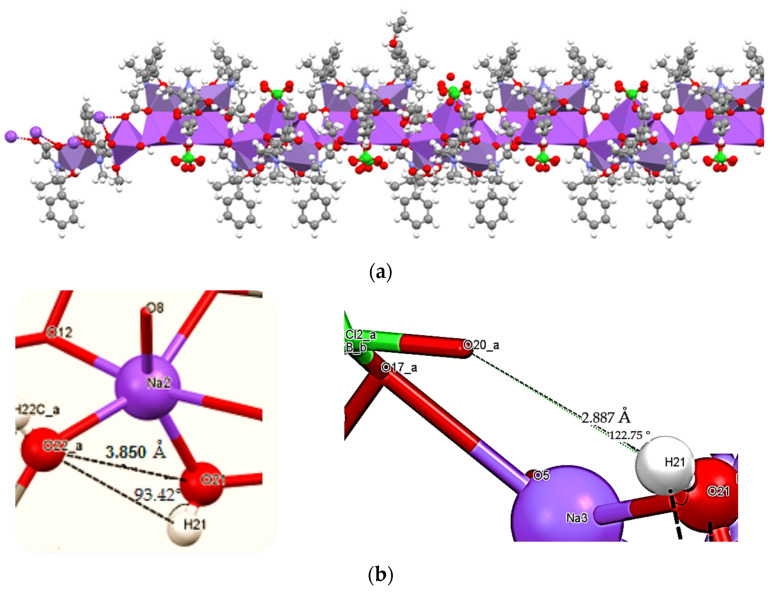
(**a**). Packing structure of compound **2** corresponding with intermolecular interactions, (**b**) C–H…O (perchlorate) and C–H…π hydrogen bonds.

Each crystallographically unique Na^+^ ion (two (Na1 and Na4) in special positions and two (Na2 and Na3) in general positions) is effectively surrounded by six O atoms (from the carboxylate, hydroxyl, perchlorate, and methanol groups), excluding the minor components of the disorder. The shortest Na^+^…Na^+^ distance is 3.339(3) Å [20], and the Na–O distances range from 2.320(5) Å (Na4–O11) to 3.048(6) Å (Na3–O7). The connection of the Mn[S,S(+)Hcpse]_2_ units to the {(NaClO_4_)_2_ (NaOH)}*_n_* chains is realized through the O7 → O12 µ_2_-carboxylate oxygen atoms, simultaneously bridging the Na1 → Na4 atoms and contributing to their distorted octahedral geometry. The octahedral geometry is completed by unique and symmetry-equivalent O13 (Cl1 perchlorate group) for Na1, hydroxy O21 and methanol-coordinated O22 solvent for Na2, and the second perchlorate O17 group (Cl2) for Na3 and Na4, resulting in the generation of repeated—Na—O—Na—O—cores with an up–down alternate of Mn[S,S(+)Hcpse]_2_ and ClO_4_. Owing to the disorder, the second perchlorate group has two µ_2_-coordination modes between Na3 and Na4, µ_2_-monodentate (Cl2 → O20) and µ_2_-bidentate (Cl2B → O20B). Adjacent pairs of the phenyl and methyl groups create cavities, which are filled with solvent molecules and hydration water, (Figure 6a). The 1D chains run along the vector [001], with the phenyl and methyl groups as pendants toward the outside surface of the endless chain, forming a lipophilic surface. Pairs of chains flowing in opposite directions are weakly connected by C–H…O (perchlorate) and C–H…π hydrogen bonds (Figure 6b) [23].

The powder X-ray diffraction pattern of compound **2** was compared with the analogous enantiomer. They present similar peaks, which correspond to the planes [1, 1, −1], [1, 1, 0], [1, 1, −2], [2, 2, −1], [1, 3, 0], indicating that these compounds can be considered isomorphous. The circular dichroism spectra for these complexes are shown as mirror images with opposite cotton effects. This behavior suggests a similar structure in solution for both enantiomers. The manganese compounds have three absorption bands, one at 565 nm and two CT bands at 356 nm and 461 nm [24] compound **2** (−,−,+) and compound enantiomeric compound (+,+,−). The first transition observed in the CD spectra ν_1_, is associated with an electronic transition for Mn^3+^. The isostructural properties of both compounds are shown in Supplementary Table S5.

### 3.3. Electrochemical Studies of the AO Biomimetic Model

Electrochemical investigations were carried out on a pair of chiral H_2_cpse ligands, two trinuclear Cu^2+^ complexes (which are enantiomeric compounds), and their racemic complex (referred to as compound **1**), as detailed in Table 4. The experiments were performed in degassed methanol due to the limited solubility of the ligands and their copper complexes in other solvents. The cyclic voltammograms of (+) S,S-H_2_cpse and (+) R,R-H_2_cpse exhibited a single irreversible oxidation peak with Epa values of 0.926 and 0.927 V, respectively. Previous work by Lloyd et al. in 2011 exclusively discussed the Cu^2+^/+ redox couple for chiral and achiral derivatives of [bis(picolyl)amino]acylglycine ethyl ester and [bis(picolyl)amino]acylphenylalanine methyl ester complexes of Cu^2+^. These complexes have square pyramidal or distorted octahedral geometries, with N_3_O coordination of the amino acid ligands, completed by additional ligands like acetonitrile, oxygen, and chloride. These studies employed similar conditions (methanol with 0.1 M tetrabutylammonium hexafluorophosphate as the supporting electrolyte) and a relatively limited electrochemical window of between −0.4 and 0.2 V vs. NHE.

Ligands not coordinated to metals lacked electrochemical data, and no redox peaks were evident in the voltammograms of copper complex compounds. Comparable electrochemical assessments were performed on trinuclear copper complexes, namely, [Cu_3_(S,S(+)-cpse)_3_]∙8.5H_2_O and [Cu_3_(R,R(-)-cpse)_3_]∙8.5H_2_O, where the H_2_cpse ligands were doubly deprotonated. These trinuclear complexes’ voltammograms lacked ligand-based oxidation peaks. The voltammogram of [Cu_3_(S,S(+)-cpse)_3_]∙8.5H_2_O displayed three reduction peaks and two oxidation peaks. The two reduction peaks at −1.399 and −0.982 V corresponded to Cu^+^ reduction to Cu0. The third reduction peak at 0.430 V corresponded to Cu^2+^ reduction to Cu^+^, whereas the oxidation peak at 0.145 V corresponded to Cu0 oxidation to Cu^+^, and the peak at 0.414 V corresponded to Cu^+^ oxidation to Cu^2+^. The Cu0/+ oxidation peak exhibited more width than anticipated, suggesting similar but non-equivalent electrochemical behavior of the three Cu^2+^ centers.

For [Cu3(S,S(+)-cpse)3]∙8.5H_2_O, two reduction peaks appeared in the voltammogram, whereas [Cu3(R,R(-)-cpse)3]∙8.5H_2_O showed a broad oxidation peak at 0.193 V due to the overlap of Cu^+^ stripping and Cu^2+^ oxidation peaks. These examinations on the trinuclear cpse complexes highlighted distinct properties among the three Cu^2+^ centers influenced by ligand chirality. The racemic mixture exhibited a mixed electrochemical response, interplaying enantiomerically pure compounds. Reduction peaks at −1.389 and −0.872 were attributed to Cu^+^ reduction to Cu0. The peak at −0.445 V corresponded to Cu^2+^ reduction to Cu^+^, and the peak at −0.410 V corresponded to Cu^+^ oxidation to Cu^2+^. These electrochemical characteristics in the racemic compound further underscored the presence of metal centers with analogous chemical properties yet differing electrochemical behavior. This aligns with the presence of coexisting enantiomers in the crystalline structure, as corroborated by Lloyd et al. in 2011 (Table 4, Supplementary Table S6).

### 3.4. Electrochemical Studies of the Catalase Biomimetic Model

CV experiments were also conducted to analyze the electrochemical behavior of compound **2**. The results are dependent on the starting direction of the CV scan. When the scan began in the negative potential direction, no peaks were observed. In contrast, when the scan began in the positive potential direction, an oxidation peak was observed (Ep_Ia_ = 0.61 V), which can be attributed to oxidation of the metal center. In the inverse scan, three reduction peaks were observed (Ep**_Ic_** = 0.47 V, Ep**_IIc_** = 0.06 V, and Ep**_IIIc_** = −0.6 V), where peak **Ic** is related to the reduction of the oxidation products of peak **Ia** back to the original species, as shown in Table 5. Owing to the current intensity in peak **IIc** being considerably lower than that in peak **Ia**, the redox wave in **Ia**–**Ic** corresponds to at least one electron transfer followed by a chemical reaction, where the chemical step could be the result of either bond breaking or bond formation. Conversely, peaks **IIc** and **IIIc** should correspond to the reduction in some intermediates produced in the chemical reaction, because they did not appear when the CV scan began in the negative direction. When the CV was extended across the entire potential window of the system (from −2.5 to 2.0 V), no new electrochemical signals were detected (19). For the enantiomer compound, a similar performance was observed as in the *oxidase ascorbate* model; the presence of possible intermediaries and electrochemical differences in the behavior of metal ions..

**Figure 7:**
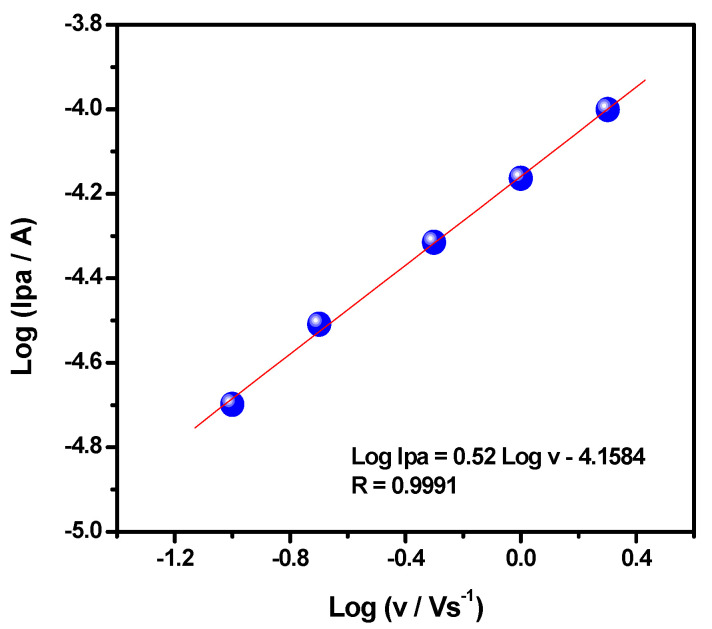
Linear dependence of the oxidation peak current (Ipa) on the scan rate (v).

In order to estimate the number of electrons transferred during the oxidation process, a comparison of the electrochemical behavior of compound **2** and a one-electron transfer system, such as ferrocenecarboxaldehyde (Fc–CHO), was made [25]. The oxidation peak current of compound **2** (20.5 μA) had the same order of magnitude as the oxidation peak current of Fc–CHO (29.3 μA), implying that compound **2** underwent one-electron oxidation as well. The small difference (~9 μA) between these two anodic currents could be attributed to the low diffusion coefficient of compound **2**, which is due to its large size, and the fact that this complex did not dissolve completely in the used solvent. The peak-to-peak separation (∆Ep = Ep_Ia_ − Ep_Ic_) at 0.1 Vs^−1^ is 140 mV, suggesting that the electron transfer process is slow. To corroborate this suggestion, an analysis of the electrochemical behavior with respect to the scan rate was conducted. It is necessary to investigate whether the electroactive species diffuse from the solution or are adsorbed on the electrode surface before conducting any mechanistic analysis [23,24,25,26]. Thus, the variation of the anodic peak current with respect to the scan rate (Log Ipa/Log v plot) was plotted, as shown in Figure 7 The slope of this plot was 0.52, indicating that the electron transfer was controlled by diffusion. Consequently, no species were adsorbed during the potential scan. It can be observed that as the scan rate increases, the oxidation potential of peak **Ia** becomes more positive, whereas the reduction peak **Ic** becomes slightly negative. Thus, the higher the scan rate, the larger the Ep, which is in agreement with a slow electron transfer rate. The sluggishness of the electron transfer step is supported by the slope of the Epa/Log v plot (∂Epa/∂Log v = 0.066 V). Thus, when this value was used in the (∂Ep/∂Log v) vs. Log k_s_ working curve, where k_s_ is the heterogeneous electron transfer constant, the value of k_s_ for the system under investigation was observed to be less than 10^−1^ cm∙s^−1^. Additionally, the fact that the current for peaks **IIc** and **IIIc** decreases as the scan rate increases supports the assumption that these signals correspond to the reduction of intermediates produced in the chemical reaction that follows the electron transfer step. An interesting aspect that can be deduced from the electrochemical study is the fact that because the starting molecule possesses two redox centers, the presence of only one oxidation peak implies that this species has a single oxidation state, which may be due to the dissociation of compound **2** into two metallic complexes in the solution state (Supplementary Tables S7–S9).

### 3.5. Magnetic Properties of the AO Biomimetic Model

**Figure 8:**
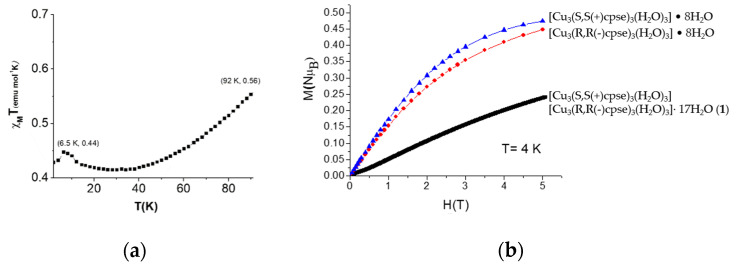
Magnetism analyses of compound **1**. (**a**) Temperature dependence of χ_M_T vs. T for compound **1** and (**b**) magnetization of the trinuclear compounds at variable magnetic fields at T = 4 K.

The pure enantiomers had vastly different magnetic properties from those of the trinuclear copper (II) compound, **1**, which was obtained from a racemic mixture of the mononuclear compounds. The χ_M_T vs. T (K) curve of compound **1** exhibits a typical antiferromagnetic coupling behavior from T = 92 to 30 K [26]. It exhibits a minimum around 30 K and χ_M_T = 0.48 emu mol^−1^ K. Furthermore, there is an abrupt increase as the temperature decreases, reaching a peak of χ_M_T = 0.44 emu mol^−1^ K at T = 6.5 K. The decrease in χ_M_T at T = 6.5 K is due to saturation effects [27] and strong antiferromagnetic coupling at T = 298 K. For this system, a mixture of oxidation states for Cu1–Cu2–Cu3 based on thermal activation is suggested. Three states are possible: a quartet (│3/2,1>) and two doublets (│1/2,1>). The states considered for the range 30 K < T < 92 K include │1/2, 1> 92 K and │3/2, 1>, as shown in Figure 8a. For the pure enantiomer and racemic compounds, external fields between 1 T and 5 T were measured. There are structural differences between these compounds. The racemic mixtures have a double water molecule and more directional intermolecular interactions than their respective pure enantiomers. The amount of water outside the coordination sphere is believed to significantly influence the paramagnetic–antiferromagnetic transition (Figure 8b).

### 3.6. Magnetic Properties of the Catalase Biomimetic Model

**Figure 9:**
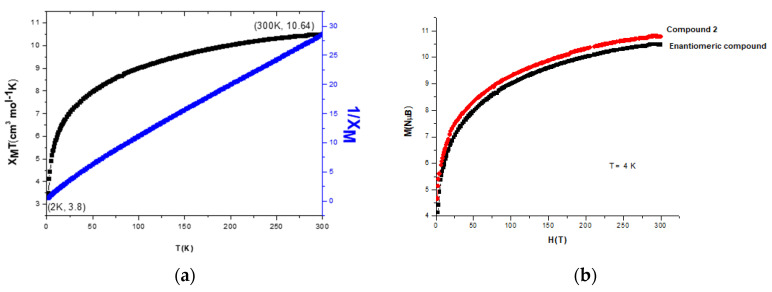
Magnetism analyses of compound **2.** (**a**) Temperature dependence of χ_M_T vs. T and 1/χ_M_T vs. T for compound **2** and (**b**) magnetization of compound 2 and its enantiomeric compound at T = 4 K.

For compound **2**, the χ_M_ vs. T(K) and 1/χ_M_ vs. T(K) plots exhibit a typical antiferromagnetic coupling behavior at 3 KOe (Figure 9a). In χ_M_T vs. T(K), χ_M_T decreases as the temperature decreases. The value of χ_M_T at T = 300 K is 10.64 cm^3^ K mol^−1^, which corresponds to two manganese (II) ions (S = 5/2), which is consistent with a dimer (it decreases to T = 2 K, χ_M_T = 3.80 cm^3^ mol^−1^ K) [27]. The inverse molar susceptibility suggests an antiferromagnetic behavior when the parameters obtained by fitting the Curie–Weiss plot are between 300 K and 2 K, θ = −16.58 K, and C = 0.0917. The corresponding molar magnetic susceptibility is represented using the Heisenberg model (H = −2JS1·S2, S1 = S2 = 5/2) and Equation (3).

(3) $$\chi_{M}=\frac{{2N\beta }^{2}g^{2}}{3\mathrm{KT}}x^{}\;\;\frac{e^{2x}+{5e}^{6x}+{30e}^{20x}+{55e}^{30x}}{1+{3e}^{2x}+{5e}^{6x}+{7e}^{12x}+{9e}^{20x}+{11e}^{30x}}$$

where, N, g, β, and x are Avogadro’s number, the g factor, Bohr’s magneton, and J/KT, respectively. The best-fit parameters (coefficients with 95% confidence bounds) obtained using nonlinear regression analysis are g = 2.58 and 2J = 20.88 cm^−1^, indicating an excellent data fit and moderate antiferromagnetic ordering. The X-band EPR spectra of a polycrystalline sample of compound **2** in the solid state exhibited an isotropic featureless signal centered at g = ~2.0 and typical signals for the axial geometry around the manganese (II) ions. However, the EPR spectra in the solution state at 2.03 mM and T = 110 K exhibited the non-Kramers doublet near 1500 G. This behavior is attributed to manganese (III), which has four unpaired electrons and a Jahn–Teller distortion, resulting in a 5Eg ground state [28]. Consequently, this indicates that the binuclear unit dissociates in the solution state, resulting in the stabilization of the manganese (III) species [25]. In Figure 9b, the magnetic behavior of the coupled enantiomers can be observed. There are a few magnetic differences between the compounds that would affect biologic activities, as will be suggested in the next section.

### 3.7. Assessment of the Activity of the AO Biomimetic Model

**Figure 10:**
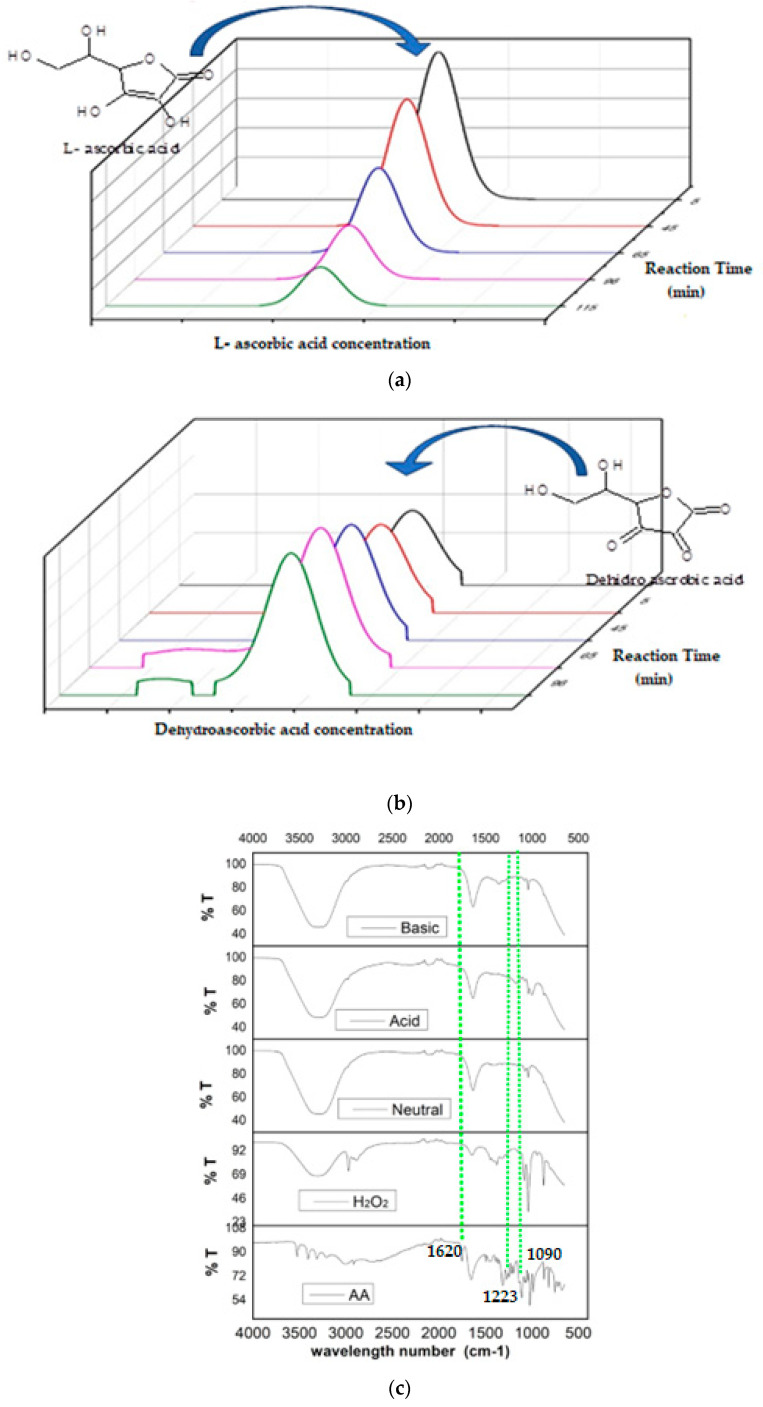

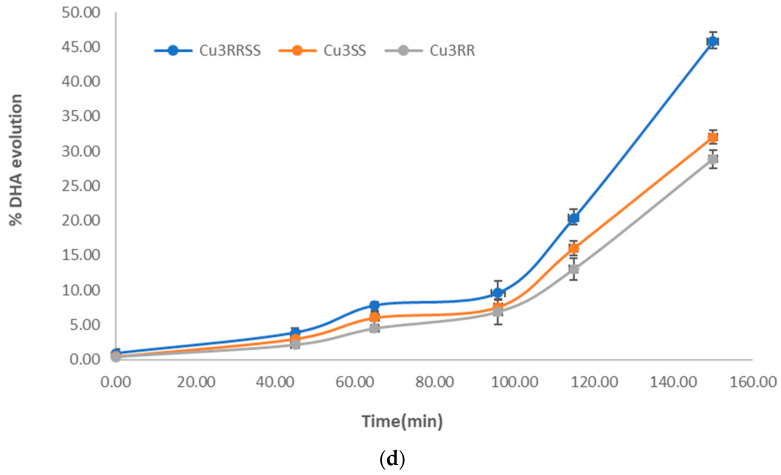
(**a**) Performance for L-AA oxidation by compound **1**, (**b**) performance of dehydroascorbic acid produced using the AO biomimetic model, (**c**) IR spectra for oxidation evolution for L-AA and (**d**) %DHA evolution for compound **1** and pure enantiomer (5 ppm) and L-AA (100 ppm).

The structural resemblance between the AO enzyme’s active center and the structural model presented in this study offers an initial insight into the catalytic properties of compound **1** [21,22]. Both AO and compound 1 possess a Cu^2+^ trinuclear center, situated at similar distances (approximately 3.5 Å). These compounds contain electron-donating atoms, nitrogen in the amino group and oxygen in the carboxyl group [29]. To investigate compound **1**’s present biomimetic properties, the degradation of L-ascorbic acid (L-AA) was used as a model to compare it to AO’s activity. The results showed a direct correlation between the decrease in L-AA concentration and the increase in the concentration of trinuclear compound **1**. The degradation of L-AA at neutral pH was comparable to the basic pH, indicating that pH variations did not significantly affect the degradation process. Analyzing the FT-IR spectra, it was observed that the disappearance of the band corresponding to the oxidation of secondary alcohols in L-AA was linked to the formation of dehydroascorbic acid. The oxidant model H_2_O_2_ was used to better understand the product’s performance. When comparing racemic and enantiomerically pure compounds, a linear trend in L-AA degradation to dehydroascorbic was noted. The racemic compound and the pure trinuclear compound at 5 ppm demonstrated similar efficiency in % DHA (dehydroascorbic acid) evolution for an initial ascorbic acid concentration of 100 ppm. However, slight differences between enantiomers were observed due to electrochemical analyses, showing asymmetric catalysis. The catalyst’s chiral structure allows it to exist in two different enantiomeric forms, selectively promoting the formation of a specific stereoisomer. In contrast, a weak bond catalyst was observed for the racemic compound. The catalyst formed weak bonds with substrates, enabling precise spatial orientation and specific stereoisomer formation. Hydrogen bonds were particularly influential in favoring ascorbic acid degradation. The presence of water arrangements between trinuclear isomers in the racemic compound significantly contributed to substrate degradation. The study suggested that the racemic compound’s super magnetic exchange path between trinuclear centers led to a greater number of redox centers, enhancing the conversion of ascorbic acid to dehydroascorbate. Surprisingly, the racemic compound exhibited catalytic activity and even outperformed its pure enantiomers in this specific case due to the exchange between isomer units. In many chemical reactions, racemates are produced in large quantities because the reactions are not stereoselective. This study demonstrates how racemates can be utilized as a catalyst for ascorbic acid degradation, showing better efficiency than their pure enantiomers. Industrial use of racemates as catalysts can be challenging due to the required selectivity and the need for efficient and cost-effective catalysts. However, recent advances in this field have led to the development of new catalysts and strategies for the enantiopure production of chemical compounds or the use of racemates as catalysts (Figure 10a–d).

### 3.8. Assessment of the Activity of the Catalase Biomimetic Model

The oxidation activity of compound **2** can be represented by Equation (4).

(4) $${2H}_{2}O_{2(l)}\to 2H_{2}O_{\left(l\right)}+O_{2(g)}$$

**Figure 11:**
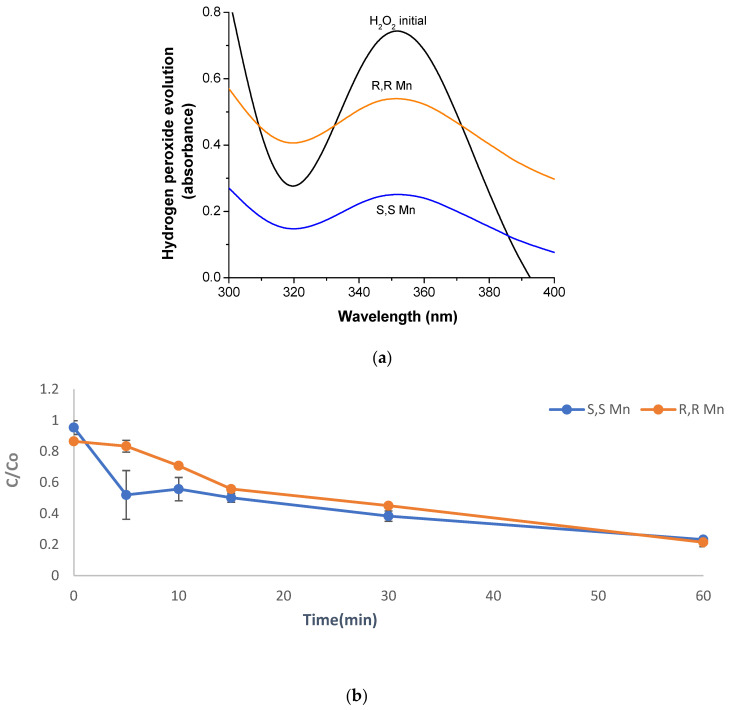
(**a**) Spectra corresponding to hydrogen peroxide reduction by compound **2** and its enantiomer (reaction time = 10 min), (**b**) kinetic degradation of hydrogen peroxide (reaction time = 0–60 min).

The electrochemical measurements and magnetic properties indicate that there is an electron transfer between the metallic centers in these units, resulting in the oxidation of Mn^2+^ to Mn^3+^. This transfer of change reduces the available catalytic sites for hydrogen peroxide oxidation. Additionally, the manganese (III) ion exhibits absorption in the visible region due to an electronic transition in the octahedral geometry (t2g3 eg → t2g2 eg2). On the other hand, manganese (II) does not show transitions in the high-spin configuration for this geometry, indicating structural distinctions between its solid and solution states. This observation allowed us to assess catalytic activity via hydrogen peroxide oxidation and UV–Vis spectroscopy. The catalytic performance of the manganese-based biomimetic complex was tested by measuring the reduction of hydrogen peroxide using the iodometric method and monitoring it spectrophotometrically at 350 nm. When using Mn complex (5ppm) and H_2_O_2_ (50 ppm), Figure 11a shows a reduction of 27% in the initial hydrogen peroxide concentration after 5 min of reaction, which is attributed to the catalytic activity of the R,R manganese compound. In contrast, the S,S isomer as a catalyst exhibits a greater decrease of 64.7% in the initial hydrogen peroxide concentration at the same time. This difference in reaction progress between the enantiomers suggests that stereochemistry ligands play a role in affecting the bridge between metal centers. The electronic exchange, which is observable in solution, is limited by the S,S isomerism, which leads to a better disposition of the substrate, in this case, hydrogen peroxide. However, after 60 min, the catalytic conversion stabilizes, and the enantiomeric effect diminishes, as shown in Figure 11b. The results demonstrate that compound **2** serves as a reliable structural and functional biomimetic model for mangano catalases, exhibiting mixed oxidation states between metal centers. The intramolecular redox process from manganese (III) to manganese (II) enables the oxidation of organic molecules at a potential of 1.559 V. The bridge between manganese metal centers typically consists of hydroxy groups, which, in an aqueous solution, can deprotonate and facilitate oxidation processes. This model provides an approximation of how amino alcohols can act as enantioselective biomimetic models for catalase and peroxidases. However, further investigation is required to delve deeper into the evolution of the mechanism, which can be addressed in subsequent studies.

Table 6 shows a comparison of different models of ascorbic oxidase and catalase that have been reported in previous studies. In the case of ascorbic oxidase, the ligand coordinates cobalt(III) through nitrogen donors in the equatorial positions, resulting in the loss of one oxime proton and the formation of an intramolecular hydrogen bond. The oxidase’s catalytic is influenced by both the tetragonal splitting and the Lewis- acidity of cobalt(III), which are determined by the natura of coordinated axial ligands. This behavior is significant because the biomimetic models used in this research involve a water molecule coordinated in the apical position, serving as a pathway between metal ions and subsequent electronic transitions. This suggests that oxidation and reduction processes for the metal ions occur via hydrogen bonding. The chiral properties of these models can be adjusted by manipulating the spatial arrangement of the ligands, which, in turn, influences the catalytic properties. On the other hand, for the catalase model, stability in the equatorial zone is crucial, and the chelate effect allows for availability of the axial positions around the metal center. The oxidation or reduction of the substrate, hydroxide peroxide, requires an electronic interchange between manganese ions. The presence of binuclear compounds Mn(III)-Mn(II) facilitates the oxidation or reduction in the substrate. Electrochemical analyses indicate that this electronic exchange occurs in aqueous solutions, leading to the subsequent oxidation and reduction of hydroxide peroxide.

## 4. Conclusions

Two novel biomimetic compounds (designated as **1** and **2**) were successfully synthesized to mimic the active centers of two important enzymes, AO and catalase. Compound **1** displayed antiferromagnetic coupling in the trinuclear systems, exhibited optical properties characteristic of a racemic compound, and possessed a structure resembling the active center of the AO metalloenzyme. On the other hand, compound **2** showed antiferromagnetic coupling in its metal ions, demonstrated pure enantiomer optical properties, and had a polymeric structure with an asymmetric unit, similar to the active center of the catalase metalloenzyme. The AO model (compound **1**) was found to be associated with stereoselective processes. The study evaluated the activity of both pure enantiomers and racemic mixtures at concentrations of 5 ppm (catalyst) and 100 ppm (AA). The magnetic and electronic interactions between metal centers in racemic compounds allowed the stabilization of a six-copper arrangement, which was crucial for the catalyst via hydrogen bonding. On the contrary, the catalase biomimetic model (compound **2**) exhibited high catalytic activity in the oxidation of hydrogen peroxide to oxygen and water. However, the electronic transfer between its metal centers in an aqueous medium led to a decrease in catalytic activity. The structure of compound **2** in a solution state needs improvement compared to its solid-state form. The findings suggest that these biomimetic models could find potential applications in industrial systems where substrates require oxidation with compounds containing environmentally friendly metal centers like manganese and copper. Nonetheless, further studies on reuse cycles and toxicological analyses of the aqueous solution after the catalytic action of compounds **1** and **2** are necessary in future works to ensure their practical viability.

## Figures and Tables

**Table 1:** Crystal data and structure refinement of compounds **1** and **2**.

Chemical Formula	2(C_36_H_51_N_3_O_12_Cu_3_) 17(H_2_O) (1)	C_54.15_ H_87.30_ Cl_2_Mn_2_N_4_Na_3_O_26_ (2)
Formula weight (g mol^−1^)	2123.14	1460.13
Crystal size (mm^3^)	0.20 × 0.15 × 0.15	
Crystal color	Blue	dark purple
Crystal system	Trigonal	Monoclinic
Space group	R-32 c	C2
Unit cell dimensions		
a (Å)	15.5811 (2)	21.5749 (8)
	15.5811 (2)	
	72.7841 (9)	
b (Å)	15.5811 (2)	18.8865 (5)
c (Å)	72.7841 (9)	17.6692 (6)
α (°)	90.0	90.0
β (°)	90.0	113.624 (4)
γ (°)	120.0	90.0
V (Å^3^)	15302.5 (2)	6596.4 (4)
Z	6	4
D_calc_ (g/cm^3^)	1.382	1.409
F (000)	3897	3061.0
Temp (K)	293 (2)	130
θ range (°)	1.87–27.489	3.3855–29.4984
Index range	−20 ≤ *h* ≤ 20	−27 ≤ *h* ≤ 24
	−21 ≤ *k* ≤ 21	
	−*50* ≤ *l* ≤ *50*	
	−20 ≤ *k* ≤ 20	−25 ≤ *h* ≤ 24
	−21 ≤ *k* ≤ 21	
	−*50* ≤ *l* ≤ *50*	
	*−78* ≤ *l* ≤ *94*	−24 ≤ *l* ≤ 21
	−21 ≤ *k* ≤ 21	
	−*50* ≤ *l* ≤ *50*	
Reflections measured	45418	27019
Independent reflections	3897	15140
Reflections	3146	15139
R_int_	0.0679	0.0605
R	0.0466	0.0621
R_w_	0.1346	0.1438
S	1.04	0.926
∆ρ Maximum (e/Å^3^)	0.78	------
∆ρ Minimum (e/Å^3^)	−0.43	------
Flack parameter	------	------
Rint=∑Fo2−Fo2/∑Fo2,R1=∑Fo−Fc/∑Fo,wR2=∑wFo2−Fc22/∑wFo2212		

**Table 2:** Selected angles and distances for compound **1**.

Atoms	Angles (°)
O5–Cu1–N1	82.4 (1)
N1–Cu1–O3	84.5 (1)
O4–Cu1–O4A	166.7 (1)
O5–Cu1–O4	97.0 (1)
N1–Cu1–O4	106.1 (1)
**Atoms**	**Distances (** **Å)**
Cu1A-O7	1.985
Cu1-O17	2.322
Cu1-O1	1.932
Cu1-N4	2.018
Cu1B-O7	1.947

**Table 3:** Hydrogen bonds for compound **1**.

D–H...A	d(D–H)	D(D...A)	<(DHA)
O17—H171···O8 ^iii^	0.83	2.732 (7)	165
O17—H172···O17 ^ii^	0.83	2.755 (7)	155
O21—H211···O18 ^i^	0.82	2.972 (7)	175
O20—H201···O21	0.82	2.821 (7)	179
O18—H181···O7	0.87	2.771 (7)	173
O19—H191···O20 ^iv^	0.84	2.769 (7)	178
O20—H202···O20 ^iii^	0.82	3.062 (7)	139

Symmetry codes: (iii) x − y + 1/3, −y + 2/3, −z + 7/6; (ii) −x + y, −x + 1, z; (i) −y + 1, x − y + 1, z; (iv) −y + 2/3, −x + 4/3, z − 1/6.

**Table 4:** Electrochemical potentials (vs. NHE) from the CV studies of the tested complexes in methanol.

Compound	*E*p_a_ (V)	*E*p_c_ (V)	Δ*E* (V)	*E*_1/2_ (V)
(+)*S,S*-H_2_cpse	0.926	—	—	—
(-)*R,R*-H_2_cpse	0.927	—	—	—
[Cu_3_(*S,S*(+)-cpse)_3_]∙8.5H_2_O	0.145 ^a^ 0.414 ^b^	−1.399 ^a^, −0.982 ^a^, −0.430 ^b^	1.254 ^a^, 0.837 ^a^, 0.844 ^b^	−0.627 ^a^, −0.415 ^a^, −0.008 ^b^
[Cu_3_(*R,R*(-)-cpse)_3_]∙8.5H_2_O	0.193 ^a^	−1.321 ^a^, −0.958 ^a^, −0.445 ^b^	1.514 ^a^, 1.151 ^a^	−0.564 ^a^, −0.383 ^a^
Compound **1**	0.152 ^a^ 0.410 ^b^	−1.389 ^a^, −0.872 ^a^, −0.445 ^b^	1.237 ^a^, 0.72 ^a^, 0.855 ^b^	−0.619 ^a^, −0.36 ^a^, −0.017 ^b^

^a^ Cu^+/0^ potential. ^b^ Cu^2+/+^potential.

**Table 5:** Electrochemical potentials (vs. NHE) from the CV studies of the tested complexes in methanol.

Compound	*E*p_a_ (V)	*E*p_c_ (V)	ΔE (V)	E1/2 (V)
Compound **2**	0.61 ^a^	0.47 ^a^, 0.06, −0.6	0.14 ^a^, 0.55, 0.01	0.07 ^a^, 0.28, 0.005
[Mn_2_(R,R(−)Hcpse)_4_(NaClO_4_)_2_(NaOH)(MeOH)]n·(EtOH)_2n_(MeOH)_n_H_2_O_n_	0.59 ^a^	0.45 ^a^, 0.09, −0.5	0.14 ^a^, 0.5, 0.09	0.07 ^a^, 0.25, 0.045

^a^ Mn^3+/2+^ potential.

**Table 6:** Oxidase models reported previously.

Oxidase Models	Oxidase Catalytic Activity (%)	[Cat] (%)	Catalase Activity (%)	[Cat] (%)
[CoLCl_2_], L = oxime [30]	40	25	----	----
[L’CoLBr]ClO_4_, L = oxime, L’ = triphenylphosphine	50	25	----	----
[CoL(SCN)(Br)], L = oxime [30]	43	25		
[Mn(TMP)OAc], L = meso-tetraphenyl-porphyrinato [31]	-----	-----	97	40
[L’’’’M1(II)M2(II) Complexes], L = Chloro, M = Mn, Fe [31,32]	-----	-----	28	----
[Cu_3_(S,S(+)cpse)_3_(H_2_O)_3_] [Cu_3_(R,R(−)cpse)_3_(H_2_O)_3_]·17H_2_O	45.5	5		
[Cu_3_(S,S(+)cpse)_3_(H_2_O)_3_]·8.5H_2_O	32	5		
[Cu_3_(R, R(+)cpse)_3_(H_2_O)_3_]·8.5H_2_O	28	5		
[Mn_2_(S,S(+)Hcpse)_4_(NaClO_4_)_2_(NaOH)(MeOH)]n·[(EtOH)_2_]_n_·[(MeOH)]_n_[H_2_O]_n_			64.7	25
[Mn_2_(R,R(+)Hcpse)_4_(NaClO_4_)_2_(NaOH)(MeOH)]n·[(EtOH)_2_]_n_·[(MeOH)]_n_[H_2_O]_n_			27	25

## Supplementary Materials

The following supporting information can be downloaded at: https://www.mdpi.com/article/10.3390/biomimetics8050423/s1. CCDC data 2110994 contains the supplementary crystallographic data for compound **1**. CCDC data 2071434 contains the supplementary crystallographic data for compound **2**. These data can be obtained free of charge via http://www.ccdc.cam.ac.uk/conts/retrieving.html or from the Cambridge Crystallographic Data Centre, 12 Union Road, Cambridge CB2 1EZ, UK; fax: (+44)1223-336-033; e-mail: deposit@ccdc.cam.ac.uk. Table S1. IR spectra for trinuclear racemic copper compound. Table S2. IR spectra for polymeric manganese compounds. Figure S3. Mass spectrum FAB+ of [Mn_2_(R,R(-)Hcpse)_4_(NaClO_4_)_2_(NaOH)(MeOH)]n·(EtOH)2n·(MeOH)nH_2_On. Table S4. Fragmentation pattern for Mass spectrum corresponding to [Mn_2_(R,R(-)Hcpse)_4_(NaClO_4_)_2_(NaOH)(MeOH)]n·(EtOH)_2_n·(MeOH)nH_2_On. Figure S5. Electrochemical analyses for compound **1**. (a). Cyclic voltammograms vs. NHE for the ligands (+)S,S-H_2_cpse (1 mM), (b). (-)R,R-H_2_cpse (1 mM), (c). [Cu(S,S(+)Hcpse)_2_] (1 mM) (d). [Cu(R,R(-)Hcpse)_2_] (1 mM), (e). [Cu_3_(S,S(+)-cpse)_3_] (1 mM), and (f). [Cu_3_(R,R(-)-cpse)_3_] (1 mM) in methanol with 0.1 M tetrabutylammonium hexafluorophosphate (n-Bu_4_NPF_6_) as supporting electrolyte. Figure S6. CD spectra for the manganese polymeric compounds. a. [Mn_2_(R,R(-)Hcpse)_4_(NaClO_4_)_2_(NaOH)(MeOH)]n·(EtOH)_2_n·(MeOH)nH_2_On and b. [Mn_2_(S,S(-)Hcpse)_4_(NaClO_4_)_2_(NaOH)(MeOH)]n·(EtOH)_2_n·(MeOH)nH_2_On. Figure S7. Cyclic voltammetry for compound **3** at ~1 mM, on glassy carbon electrode (ϕ = 3 mm), in acetonitrile containing n-Bu_4_NPF_6_ 0.1 M. (a) positive direction starting scan, (b) negative direction starting scan. Scan rate: 0.1 V s^−1^ for [Mn_2_(R,R(-)Hcpse)_4_(NaClO_4_)_2_(NaOH)(MeOH)]n·(EtOH)_2_n·(MeOH)nH_2_On. Figure S8. Comparison of the current due to the oxidation of ferrocenecarboxaldehyde 1 mM (A) and compound **3** at ~1mM (B), on glassy carbon electrode (ϕ = 3 mm), in acetonitrile containing n-Bu_4_NPF_6_ 0.1 M, at a scan rate of 0.1 Vs^−1^ for [Mn_2_(R,R(-)Hcpse)_4_(NaClO_4_)_2_(NaOH)(MeOH)]n·(EtOH)_2_n·(MeOH)nH_2_On. Figure S9. Cyclic voltammetry at different scan rates for compound **3** at ~1 mM, on glassy carbon electrode (ϕ = 3 mm), in acetonitrile containing n-Bu_4_NPF_6_ 0.1 M for [Mn_2_(R,R(-)Hcpse)_4_(NaClO_4_)_2_(NaOH)(MeOH)]n·(EtOH)_2_n·(MeOH)nH_2_On.

## References

[1] Benkovic S.J., Hammes-Schiffer S. (2003). A Perspective on Enzyme Catalysis. Science.

[2] Choi J.-M., Han S.-S., Kim H.-S. (2015). Industrial applications of enzyme biocatalysis: Current status and future aspects. Biotechnol. Adv..

[3] Franssen M.C.R., Steunenberg P., Scott E.L., Zuilhof H., Sanders J.P.M. (2013). Immobilised enzymes in biorenewables production. Chem. Soc. Rev..

[4] Gu X., Zhang Y., Xu Z.-J., Che C.-M. (2014). Iron(III)–salan complexes catalysed highly enantioselective fluorination and hydroxylation of β-keto esters and N-Boc oxindoles. Chem. Commun..

[5] Zhang Z., Schreiner P.R. (2009). (Thio)urea organocatalysis—What can be learnt from anion recognition?. Chem. Soc. Rev..

[6] Balcells D. (2016). Insight into metal-catalyzed water oxidation from a DFT perspective. Advances in Organometallic Chemistry.

[7] Phale P.S., Sharma A., Gautam K. (2019). Microbial degradation of xenobiotics like aromatic pollutants from the terrestrial environments. Pharmaceuticals and Personal Care Products: Waste Management and Treatment Technology.

[8] Marqués J., Cortés A., Pejenaute Á., Zalba G. (2020). Implications of NADPH oxidase 5 in vascular diseases. Int. J. Biochem. Cell Biol..

[9] Rodríguez Couto S., Toca Herrera J.L. (2006). Industrial and biotechnological applications of laccases: A review. Biotechnol. Adv..

[10] Kunamneni A., Plou F., Ballesteros A., Alcalde M. (2008). Laccases and Their Applications: A Patent Review. Recent Pat. Biotechnol..

[11] Messerschmidt A., Ladenstein R., Huber R., Bolognesi M., Avigliano L., Petruzzelli R., Rossi A., Finazzi-Agró A. (1992). Refined crystal structure of ascorbate oxidase at 1.9 Å resolution. J. Mol. Biol..

[12] Davey M.W., Montagu M., Van Inz D., Sanmartin M., Kanellis A., Smirnoff N., Benzie I.J., Strain J.J., Favell D., Fletcher J. (2000). PlantL-ascorbic acid: Chemistry, function, metabolism, bioavailability and effects of processing. J. Sci. Food Agric..

[13] Lloyd D., Vainikka T., Murtomäki L., Kontturi K., Ahlberg E. (2011). The kinetics of the Cu^2+^/Cu^+^ redox couple in deep eutectic solvents. Electrochim. Acta.

[14] Galicia M., González-Fuentes M.A., Valencia D.P., González F.J. (2012). The effect of substituents on the anodic oxidation of aliphatic carboxylates and the passage towards a pseudo-Kolbe reaction. J. Electroanal. Chem..

[15] Conesa A., Punt P.J., van den Hondel C.A.M.J.J. (2002). Fungal peroxidases: Molecular aspects and applications. J. Biotechnol..

[16] Hofrichter M. (2002). Review: Lignin conversion by manganese peroxidase (MnP). Enzym. Microb. Technol..

[17] Wariishi H., Valli K., Gold M.H. (1991). In vitro depolymerization of lignin by manganese peroxidase of *Phanerochaete chrysosporium*. Biochem. Biophys. Res. Commun..

[18] Ávila-Torres Y., Acosta J., Huerta L., Toscano A., González F.J., Behrens N.B. (2020). Experimental data on synthesis and characterization of chiral dinuclear manganese (II-II) compounds as biomimetic models of the active center of catalase. Data Brief.

[19] Morgan Chan Z., Kitchaev D.A., Nelson Weker J., Schnedermann C., Lim K., Ceder G., Tumas W., Toney M.F., Nocera D.G. (2018). Electrochemical trapping of metastable Mn^3+^ ions for activation of MnO_2_ oxygen evolution catalysts. Proc. Natl. Acad. Sci. USA.

[20] Valencia I., Ávila-Torres Y., Barba-Behrens N., Garzón I.L. (2014). Structural, vibrational, and electronic properties of an uncoordinated pseudoephedrine derivative and its mononuclear and trinuclear copper(II)-coordinated compounds: A combined theoretical and experimental study. J. Mol. Struct..

[21] Hadjiivanov K.I., Panayotov D.A., Mihaylov M.Y., Ivanova E.Z., Chakarova K.K., Andonova S.M., Drenchev N.L. (2021). Power of Infrared and Raman Spectroscopies to Characterize Metal-Organic Frameworks and Investigate Their Interaction with Guest Molecules. Chem. Rev..

[22] Ávila-Torres Y., López-Sandoval H., Mijangos E., Quintanar L., Rodríguez E.E., Flores-Parra A., Contreras R., Vicente R., Rikken G.L., Barba-Behrens N. (2013). Structure and magnetic properties of copper(II) and cobalt(II) coordination compounds derived from optically active tridentate ligands. Polyhedron.

[23] Steiner T. (2002). The Hydrogen Bond in the Solid State. Angew Chem. Int. Ed..

[24] Herr P., Kerzig C., Larsen C.B., Häussinger D., Wenger O.S. (2021). Manganese(I) complexes with metal-to-ligand charge transfer luminescence and photoreactivity. Nat. Chem..

[25] Fabbrizzi L. (2020). The ferrocenium/ferrocene couple: A versatile redox switch. ChemTexts.

[26] Julve M., Gleizes A., Chamoreau L.M., Ruiz E., Verdaguer M. (2018). Antiferromagnetic Interactions in Copper(II) µ-Oxalato Dinuclear Complexes: The Role of the Counterion. Eur. J. Inorg. Chem..

[27] Zhao X.G., Richardson W.H., Chen J.-L., Li J., Noodleman L., Tsai H.-L., Hendrickson D.N. (1997). Density Functional Calculations of Electronic Structure, Charge Distribution and Spin Coupling in Manganese−Oxo Dimer Complexes. Inorg. Chem..

[28] Ramidi P., Felton C.M., Subedi B.P., Zhou H., Tian Z.R., Gartia Y., Pierce B.S., Ghosh A. (2015). Synthesis and characterization of manganese(III) and high-valent manganese-oxo complexes and their roles in conversion of alkenes to cyclic carbonates. J. CO2 Util..

[29] Solomon E.I., Sundaram U.M., Machonkin T.E. (1996). Multicopper Oxidases and Oxygenases. Chem. Rev..

[30] Ramadan A.E.-M.M., Shaban S.Y., Ibrahim M.M. (2011). Synthesis, characterization, and ascorbic acid oxidase biomimetic catalytic activity of cobalt(III) oxime complexes. J. Coord. Chem..

[31] Robert A., Loock B., Momenteau M., Meunier B. (1991). Catalase modeling with metalloporphyrin complexes having an oxygen ligand in a proximal position. Comparison with complexes containing a proximal nitrogen. Inorg. Chem..

[32] Balasubramanian P.N., Schmidt E.S., Bruice T.C. (1987). Catalase modeling. 2. Dynamics of reaction of a water-soluble and non.mu.-oxo dimer forming manganese(III) porphyrin with hydrogen peroxide. J. Am. Chem. Soc..

