# Supplementary material for: Enantioselective Biomimetic Structures Inspired by Oxi-Dase-Type Metalloenzymes, Utilizing Polynuclear Compounds Containing Copper (II) and Manganese (II) Ions as Building Blocks
Source: Biomimetics (Basel). 2023 Sep 13;8(5):423. doi: 10.3390/biomimetics8050423 (PMC10527443; doi:10.3390/biomimetics8050423)
Supplement: Supplementary file 1 [file biomimetics-08-00423-s001.zip › biomimetics-2454075-supplementary.pdf]

Supplementary Information

Table S1. IR spectra for trinuclear racemic copper compound

Vibration	ν(-OH)$_{st}$	ν(CH)$_{st}$ aromatic	ν_{as}(-COO$^-$)$_{st}$	ν_s(–COO-)$_{st}$	$\Delta\nu$	ν (C-OH)$_{st}$
Ligand	3063	2988	1633	1384	-------	1041
**AO biomimetic model**	3214	2825	1615	1385	230 (Monodentade)	---

Table S2. IR spectra for polymeric manganese compounds

Vibration	ν(-OH)$_{st}$	ν(CH)$_{st}$ aromatic	ν_{as}(-COO$^-$)$_{st}$	ν_σ(–COO-)$_{st}$	$\Delta\nu$	ν *(C-OH)$_{st}$*	M-ClO$_4$
Ligand	3063	2988	1633	1384	-------	1041	-------
[Mn_2(R,R(-)Hcpse)$_4$($NaClO_4$)$_2$(NaOH)(MeOH)]$_n$·(EtOH)$_{2n}$·(MeOH)$_n$$H_2O_n$	3217	2880	1569	1443	126 (Bridge)	---	ν_2(1019) ν_3 (1082) ν_4 (927)
[Mn_2(S,S(-)Hcpse)$_4$($NaClO_4$)$_2$(NaOH)(MeOH)]$_n$·(EtOH)$_{2n}$·(MeOH)$_n$$H_2O_n$	3588	2979	1569	1414	155 (Bridge)	---	ν_2(1021) ν_3 (1092) ν_4 (932)

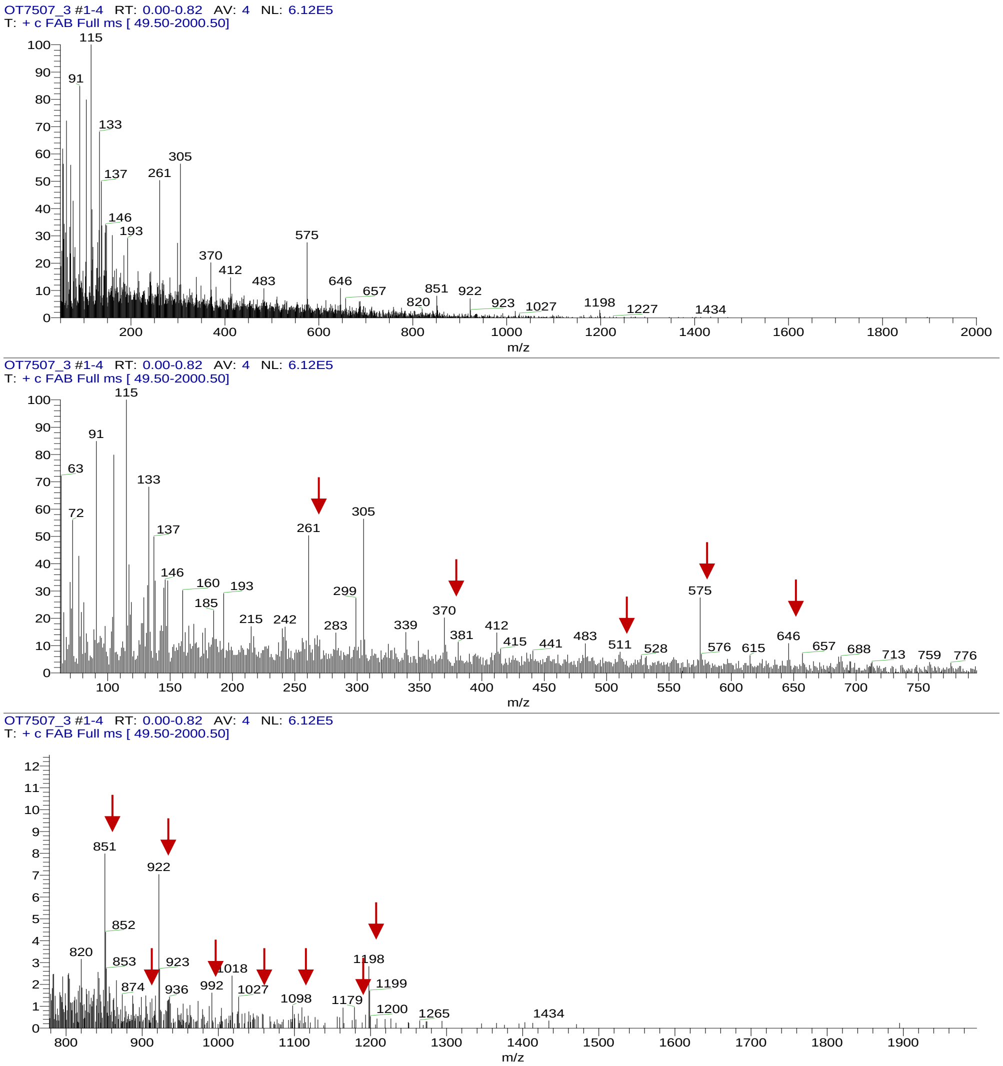

Figure S3. Mass spectrum FAB$^+$ of $[Mn_2(R,R(-)Hcpse)_4(NaClO_4)_2(NaOH)(MeOH)]n\cdot (EtOH)_{2n}\cdot(MeOH)_nH_2O_n$

Table S4. Fragmentation pattern for Mass spectrum corresponding to $[Mn_2(R,R(-)Hcpse)_4(NaClO_4)_2(NaOH)(MeOH)]_n \cdot (EtOH)_{2n} \cdot (MeOH)_n H_2O_n$

Peake	(m/z)	Assignation
1322	Dimeric unit 1323.20	$(C_{48}H_{67}Cl_2Mn_2N_4Na_4O_{22})$
1198	121.94 $(ClNaO_4)$	$(C_{48}H_{67}ClMn_2N_4Na_3O_{18})$
1179	149 $(ClNa_2O_4)$	$(C_{48}H_{67}ClMn_2N_4Na_2O_{18})$
1098	219$(Cl_2Na_2O_8)$	$(C_{48}H_{67}ClMn_2N_4Na_3O_{14})$
1027	274.9 $(Cl_2Na_3O_9CH_3)$	$(C_{47}H_{64}Mn_2N_4NaO_{13})$
992	306.92 $(Cl_2Na_3O_9CH_3)$	993.29 $(C_{46}H_{59}Mn_2N_4NaO_{12})$
922	403 $(Cl_2Na_3O_{10}C_8H_{20})$	920.18$(C_{41}H_{56}Mn_2N_4NaO_{12})$
874	445.19$(Cl_2Na_3O_{10}C_8H_{20})$	877.13$(C_{38}H_{39}Mn_2N_4NaO_{12})$
851	472.06 $(Cl_2Na_3O_{10}C_{10}H_{27})$	851.11$(C_{36}H_{39}Mn_2N_4NaO_{12})$
657	669.12 $(ClNaO_{12}MnN_2C_{25}H_{36})$	654.07 $(C_{23}H_{31}ClMnN_8O_{10})$
575	744.87$(C_{24}H_{34}MnN_2Na_3O_{15})$	574.13 $(C_{24}H_{33}ClMnN_2NaO_7)$
511	824.07 $(Cl_2Na4O_{16}MnN_2\ C_{25}H_{39})$	499.16 $(C_{24}H_{32}MnN_2O_6)$
483	839.09 $(Cl_2Na_4O_{15}MnN_2C_{26}H_{42})$	484.14 $(C_{23}H_{29}MnN_2O_6)$
412	913.13 $(Cl_2Na_4O_{17}MnN_2C_{29}H_{48})$	410.10 $(C_{20}H_{23}MnN_2O_4)$
370	928.15 $(Cl_2Na_4O_{17}MnN_2C_{30}H_{51})$	369.06 $(C_{17}H_{18}MnN_2O_4)$
115	$(C_{47}H_{63}Cl_2Mn_2N_4Na_3O_{17})$	(CH_4NaO_5)

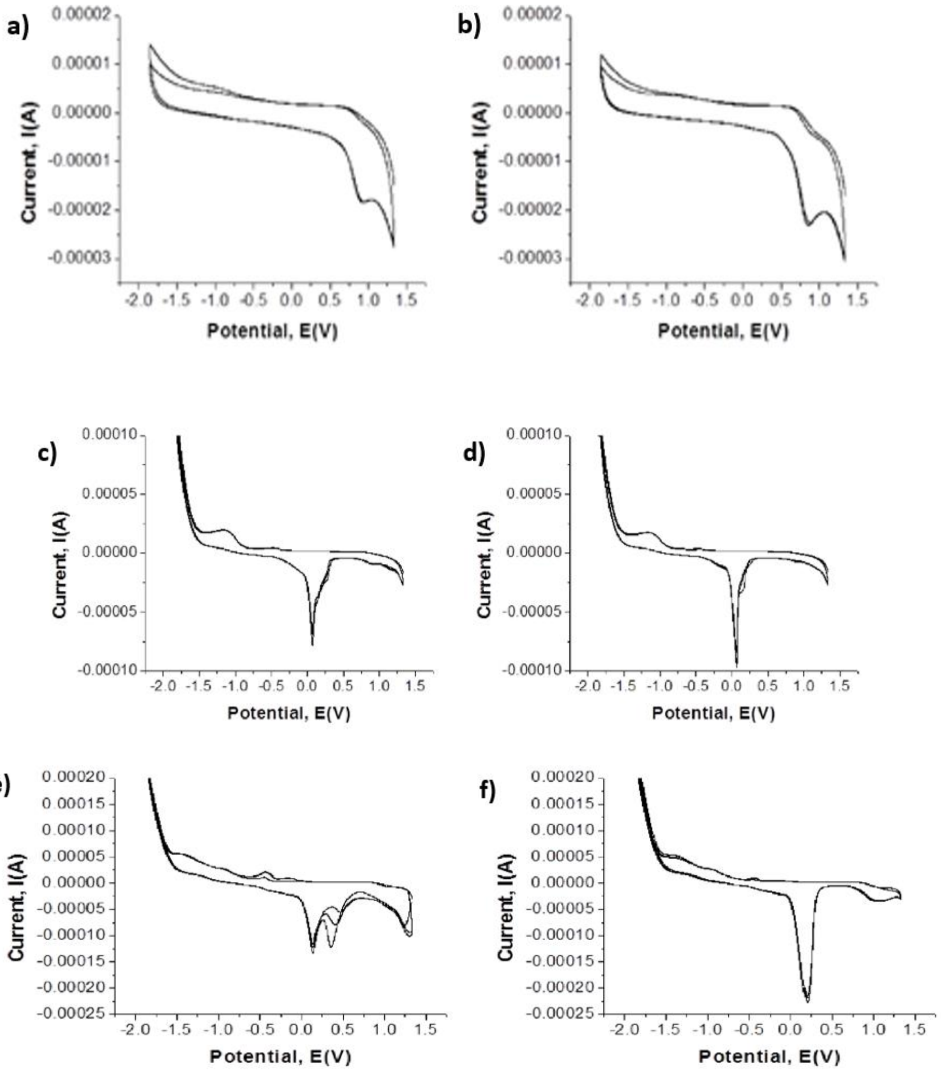

Figure S5. Electrochemical analyses for compound **1**. a). Cyclic voltammograms vs. NHE for the ligands (+)*S*,*S*-H_2cpse (1 mM), b). (-)*R*,*R*-H_2cpse (1 mM), c). [Cu(*S*,*S*(+)Hcpse)$_2$] (1 mM) d). [Cu(*R*,*R*(-)Hcpse)$_2$] (1 mM), e). [Cu_3(*S*,*S*(+)-cpse)$_3$](1 mM), and f). [Cu_3(*R*,*R*(-)-cpse)$_3$] (1 mM) in methanol with 0.1 M tetrabutylammonium hexafluorophosphate (*n*-Bu_4NPF_6) as supporting electrolyte.

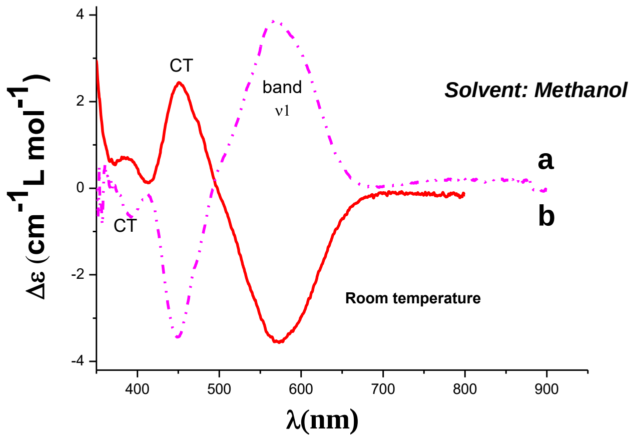

Figure S6. CD spectra for the manganese polymeric compounds.
a. $[Mn_2(R,R(-)Hcpse)_4(NaClO_4)_2(NaOH)(MeOH)]_n\cdot$ $(EtOH)_{2n}\cdot(MeOH)_nH_2O_n$ and
b. $[Mn_2(S,S(-)Hcpse)_4(NaClO_4)_2(NaOH)(MeOH)]_n\cdot$ $(EtOH)_{2n}\cdot(MeOH)_nH_2O_n$

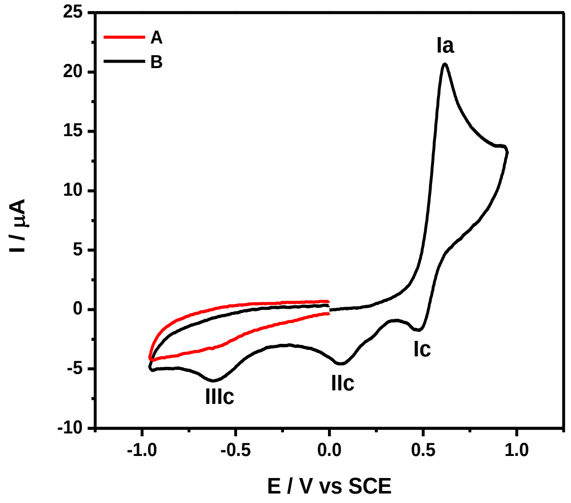

Figure S7. Cyclic voltammetry for compound **3** at ~1 mM, on glassy carbon electrode (ϕ = 3 mm), in acetonitrile containing *n*-Bu_4NPF_6 0.1 M. a) positive direction starting scan, b) negative direction starting scan. Scan rate: 0.1 V s^{-1} for $[Mn_2(R,R(-)Hcpse)_4(NaClO_4)_2(NaOH)(MeOH)]_n\cdot$ $(EtOH)_{2n}\cdot(MeOH)_nH_2O_n$

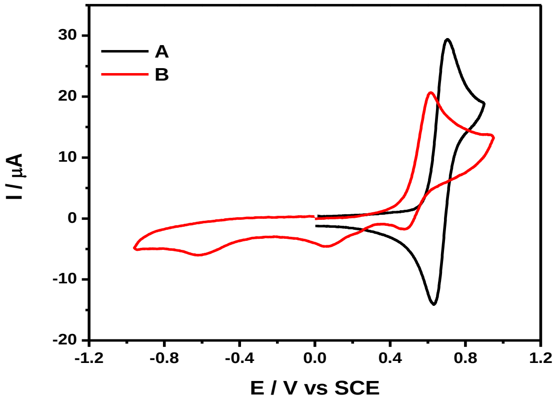

Figure S8. Comparison of the current due to the oxidation of ferrocenecarboxaldehyde 1 mM (A) and compound **3** at ~1mM (B), on glassy carbon electrode (ϕ = 3 mm), in acetonitrile containing *n*-Bu_4NPF_6 0.1 M, at a scan rate of 0.1 Vs^{-1} for $[Mn_2(R,R(-)Hcpse)_4(NaClO_4)_2(NaOH)(MeOH)]n\cdot$ $(EtOH)_{2n}\cdot(MeOH)_nH_2O_n$

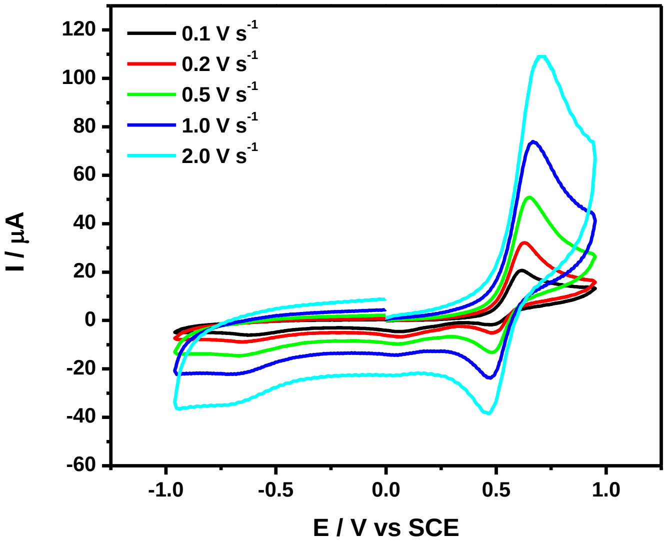

Figure S9. Cyclic voltammetry at different scan rates for compound **3** at ~1 mM, on glassy carbon electrode (ϕ = 3 mm), in acetonitrile containing *n*-Bu_4NPF_6 0.1 M for $[Mn_2(R,R(-)Hcpse)_4(NaClO_4)_2(NaOH)(MeOH)]n\cdot$ $(EtOH)_{2n}\cdot(MeOH)_nH_2O_n$
